# BladMetrix: a novel urine DNA methylation test with high accuracy for detection of bladder cancer in hematuria patients

**DOI:** 10.1186/s13148-022-01335-2

**Published:** 2022-09-17

**Authors:** Heidi Dietrichson Pharo, Marine Jeanmougin, Eirill Ager-Wick, Hege Marie Vedeld, Anne Klara Sørbø, Christina Dahl, Louise Katrine Larsen, Hilde Honne, Sara Brandt-Winge, May-Britt Five, Sara Monteiro-Reis, Rui Henrique, Carmen Jeronimo, Kenneth Steven, Rolf Wahlqvist, Per Guldberg, Guro Elisabeth Lind

**Affiliations:** 1 Department of Molecular Oncology, Institute for Cancer Research, Oslo University Hospital, The Norwegian Radium Hospital, Oslo, Norway; 2 Department of Biosciences, The Faculty of Mathematics and Natural Sciences, University of Oslo, Oslo, Norway; 3 Department of Urology, Oslo University Hospital, Oslo, Norway; 4 Danish Cancer Society Research Center, 2100 Copenhagen, Denmark; 5 Cancer Biology and Epigenetics Group, Research Center (CI-IPOP), Portoguese Oncology Institute of Porto (IPO Porto), Porto, Portugal; 6 Department of Pathology, Portuguese Oncology Institute of Porto (IPO Porto), Porto, Portugal; 7 Department of Pathology and Molecular Immunology, Institute of Biomedical Sciences Abel Salazar (ICBAS), Porto, Portugal; 8 Department of Urology, University of Copenhagen, Herlev Hospital, Herlev, Denmark; 9 Department of Cancer and Inflammation Research, Institute for Molecular Medicine, University of Southern Denmark, Odense, Denmark

**Keywords:** Biomarkers, Bladder cancer, Cystoscopy, Digital PCR, Detection, DNA methylation, Hematuria, Non-invasive detection, Methylome sequencing, Urine test

## Abstract

**Background:**

Cystoscopy is the gold standard for bladder cancer detection, but is costly, invasive and has imperfect diagnostic accuracy. We aimed to identify novel and accurate DNA methylation biomarkers for non-invasive detection of bladder cancer in urine, with the potential to reduce the number of cystoscopies among hematuria patients.

**Results:**

Biomarker candidates (*n* = 32) were identified from methylome sequencing of urological cancer cell lines (*n* = 16) and subjected to targeted methylation analysis in tissue samples (*n* = 60). The most promising biomarkers (*n* = 8) were combined into a panel named BladMetrix. The performance of BladMetrix in urine was assessed in a discovery series (*n* = 112), consisting of bladder cancer patients, patients with other urological cancers and healthy individuals, resulting in 95.7% sensitivity and 94.7% specificity. BladMetrix was furthermore evaluated in an independent prospective and blinded series of urine from patients with gross hematuria (*n* = 273), achieving 92.1% sensitivity, 93.3% specificity and a negative predictive value of 98.1%, with the potential to reduce the number of cystoscopies by 56.4%.

**Conclusions:**

We here present BladMetrix, a novel DNA methylation urine test for non-invasive detection of bladder cancer, with high accuracy across tumor grades and stages, and the ability to spare a significant number of cystoscopies among patients with gross hematuria.

**Supplementary Information:**

The online version contains supplementary material available at 10.1186/s13148-022-01335-2.

## Background

Bladder cancer accounts for around 570,000 new cases and over 210,000 deaths each year worldwide [1]. The most common symptom of bladder cancer is gross hematuria, i.e., visible blood in the urine, which is present in almost 80% of newly diagnosed patients [2]. However, hematuria may also be caused by a variety of other genitourinary conditions, and its specificity to detect bladder cancer is rather low [3]. Indeed, the incidence rate for bladder cancer among patients with gross hematuria has been estimated to 17% [4].

The standard procedure for bladder cancer detection in hematuria patients and other patients with a suspected bladder tumor is cystoscopy, which is an endoscopic examination of the bladder mucosa. Although considered the gold standard, cystoscopy is invasive, can be uncomfortable for the patients, and its diagnostic accuracy is stage- and operator-dependent [5]. It is also costly and contributes to make bladder cancer one of the most expensive cancers to manage [6]. In the USA, it is has been estimated that around 20,000 cancer cases are missed among patients with hematuria by cystoscopy each year, and that 230,000 unnecessary cystoscopies are performed [7]. Cytology, i.e., a visual inspection of urine cells under the microscope, is commonly used in combination with cystoscopy in high-risk patients. Cytology is non-invasive and has high specificity, but suffers from poor sensitivity, particularly for low-grade cancers [3, 5].

Identifying non-invasive urine biomarkers as an alternative to cystoscopy that could differentiate benign and malignant causes of hematuria would be of great benefit for both the patients and the society. Six urine tests have obtained approval from the US Food and Drug Administration (FDA) for bladder cancer detection and/or surveillance, but unfortunately they show varying performance across studies, and the accuracy is often poor for low-stage and low-grade tumors [8]. Consequently, none of these tests are currently recommended in routine clinical practice [9, 10]. In addition, a variety of urinary molecular biomarkers have been reported and reviewed elsewhere [11–13], but small and unrepresentative clinical series, absence of proper control groups, suboptimal sensitivity, in particular for low-grade tumors, and lack of validation are factors that typically prevent translation of biomarkers into clinical practice.

Aberrant DNA methylation is a frequent and early event in bladder carcinogenesis and has promising biomarker potential [14]. Still, only a handful of methylation biomarkers have been reported with both high sensitivity and specificity (> 90%) for urine-based detection of bladder cancer [15, 16].

The aim of the present study was to develop a highly accurate urine DNA methylation test for detection of bladder cancer, with the potential to reduce the number of costly and uncomfortable cystoscopy examinations for hematuria patients.

## Results

**Fig. 1:**
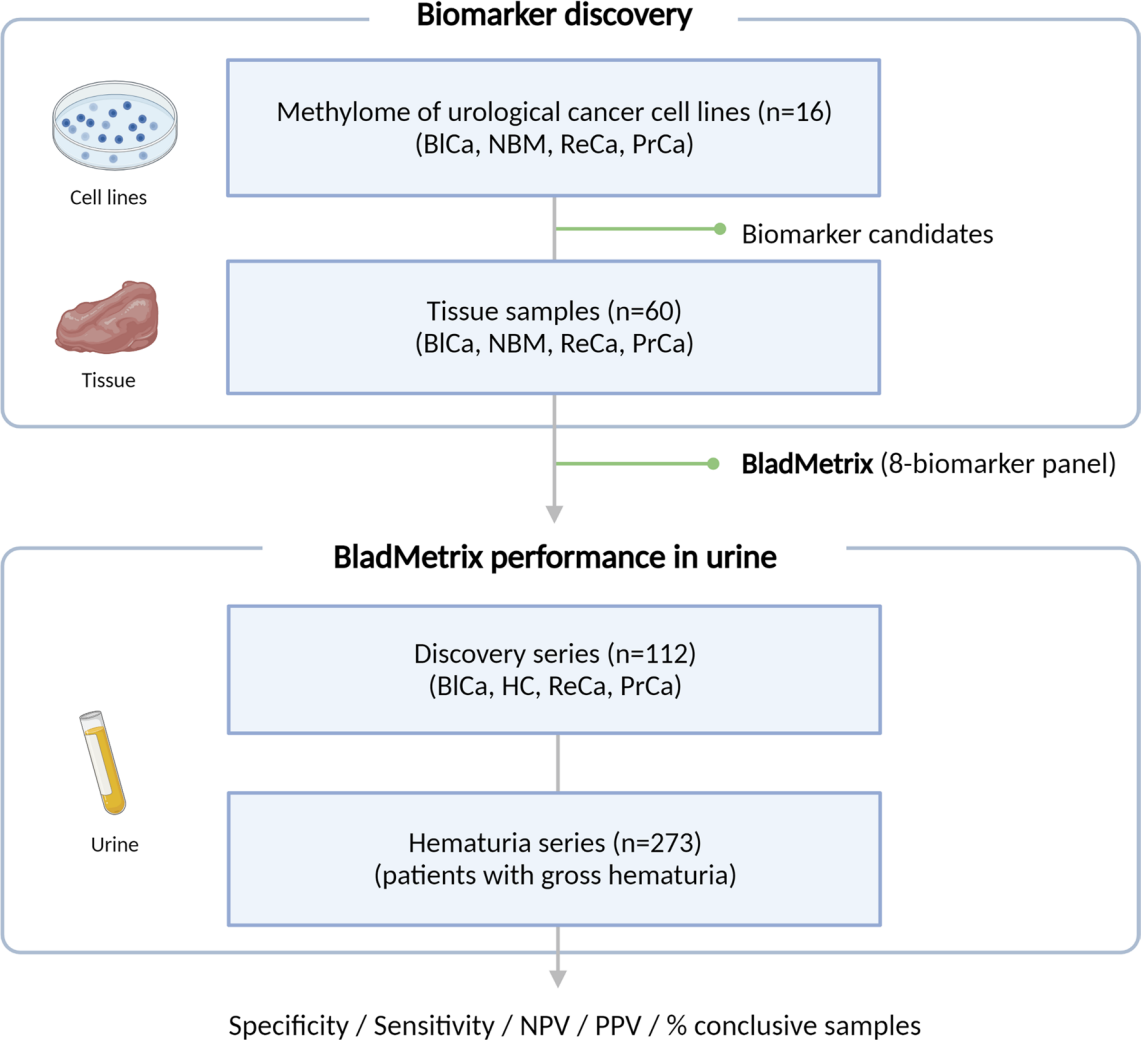
The overall strategy for biomarker discovery and evaluation of the BladMetrix test performance in urine. The figure gives an overview of the overall strategy for identification of accurate biomarkers for bladder cancer detection (upper part), and evaluation of the best performing biomarkers as a panel, i.e., the BladMetrix test, in urine (lower part). *BlCa* Bladder cancer; *NBM* Normal bladder mucosa; *HC* Healthy controls; *PrCa* Prostate cancer; *ReCa* Renal cancer; *NPV* Negative predictive value; *PPV* Positive predictive value. Figure created with BioRender.com

The overall strategy used for biomarker discovery and evaluation of biomarker performance in urine, including in a large series of hematuria patients, is shown in Fig. 1.

### Methylome-wide discovery of DNA methylation biomarker candidates

The workflow established for identification of differentially methylated regions **(**DMRs) from raw RRBS (reduced representation bisulfite sequencing) data is explained in details in Additional file 1: Methods. After quality control and trimming of the RRBS data, samples were left with on average 106 million reads (Additional file 1: Fig. S1 and Table S1). About 71% of the reads were uniquely mapped and kept for downstream analyses (Additional file 1: Fig. S2). The average coverage and mean methylation level per sample were 111.6X and 37.2%, respectively (Additional file 1: Table S1). Calculation and filtering of DMRs resulted in 214 windows fulfilling the selection criteria (Additional file 1: Fig. S1), including 64 200-bp windows and 150 1000-bp windows. Of note, the 200-bp and 1000-bp windows were overlapping substantially, indicating that the 1000-bp window strategy was possibly redundant. Overlapping windows were considered as a single biomarker candidate, resulting in 32 candidates (Additional file 1: Fig. S3 and Table S2). Almost 60% (19/32) of the candidates were located more than 1,500 bp away from the nearest transcription start site (TSS; Additional file 1: Table S2).

### Selection of the most promising biomarker candidates

A stepwise selection of the most promising biomarkers among the 32 candidates identified from methylome sequencing was performed (Additional file 1: Fig. S4). In brief, qMSP assays were designed for 28 of the 32 biomarker candidates, the four remaining candidates having too few CpG sites to allow assay design. Nineteen assays passed the quality control, all displaying high concordance with the RRBS data. Vimentin (*VIM*), identified as a promising bladder cancer methylation biomarker in our previous study [17], was included in subsequent analyses. In tissue samples (*n* = 60), the sensitivities of these 20 biomarker candidates ranged from 25 to 100%, and the specificities from 92 to 100% (Additional file 1: Table S3). When prostate and renal cancer samples were included in the control group, the specificity of the biomarkers remained high (92.5–100%; median = 97.5%; Additional file 1: Table S3). Biomarker candidates with > 50% sensitivity and a significant AUC (area under the ROC curve; *n* = 12) were considered for further analysis in urine using droplet digital PCR (ddPCR).

### BladMetrix performance in urine—the discovery series

**Fig. 2:**
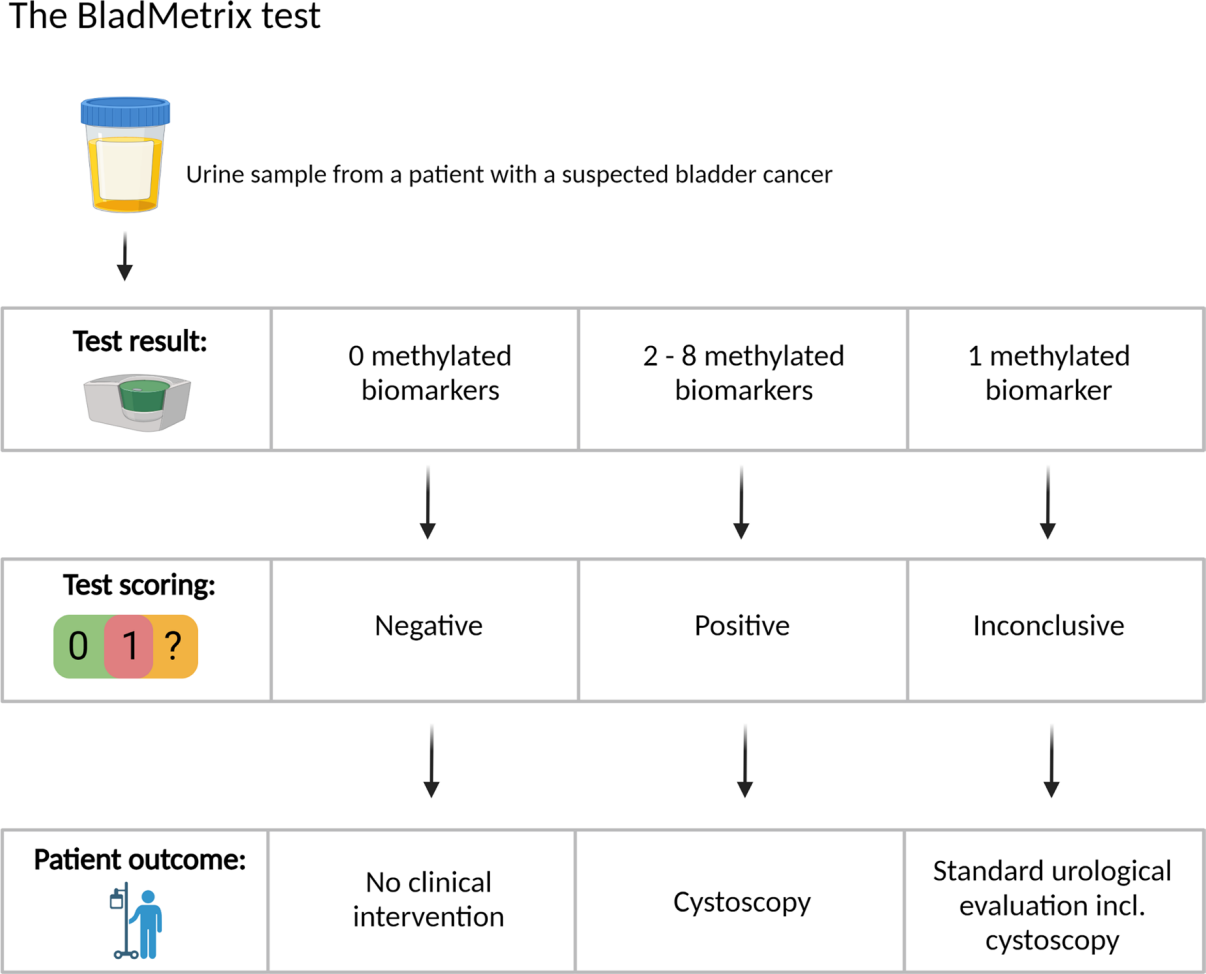
Suggested clinical use and scoring of the BladMetrix test. The BladMetrix test, which is an 8-biomarker panel, is scored negative, positive or inconclusive depending on the number of methylated biomarkers. A negative test will spare the patient for a clinical intervention, while both a positive and an inconclusive test will require the patient to undergo cystoscopy, which is in line with todays’ standard routine. Figure created with BioRender.com

Eight biomarker candidates demonstrated good technical performance using ddPCR (Additional file 1: Fig. S4). The sensitivities of the individual biomarkers in the urine discovery series (*n* = 112) ranged from 54 to 73% (Additional file 1: Table S4). The specificities ranged from 95 to 96% and the area under the ROC curves (AUCs) from 0.68 to 0.85, considering only healthy individuals in the control group (Additional file 1: Fig. S5 and Table S4). Including prostate- and renal cancers in the control group resulted in specificities ranging from 94 to 98% (Additional file 1: Table S4). Testing for potential age dependent methylation showed no significant difference when stratifying the bladder cancers in two equally sized age groups (Additional file 1: Table S5). Given an overall high performance, all eight biomarkers were combined into a panel named BladMetrix. Various approaches for defining scoring thresholds were tested, including generalized linear models using individual biomarkers as covariates or by integrating all 8 biomarkers (data not shown). Of these approaches, individual scoring of the biomarkers had the best performance. A cutoff of ≥ 2/8 methylated biomarkers was found to give the most optimal combination of sensitivity, specificity and number of conclusive test results (Additional file 1: Table S6), and was used to define a positive test. A negative test was defined as 0/8 methylated biomarkers, and samples with 1/8 methylated biomarkers were considered inconclusive. Figure 2 illustrates the suggested clinical use and scoring of the BladMatrix urine test for a patient with a suspected bladder cancer. In the discovery series, BladMetrix achieved a sensitivity of 95.7% (22/23) and a specificity of 94.7% (71/75), considering the conclusive test results, while 12.5% (14/112) of the samples were scored inconclusive (Table 1 and Additional file 1: Table S7).

**Table 1:** BladMetrix performance in the discovery and hematuria urine series

	Discovery series (*n* = 112)	Hematuria series (*n* = 273)
Conclusive patients	87.5% (98/112)	93.0% (254/273)
Sensitivity	95.7% (22/23)	92.1% (82/89)
Specificity	94.7% (71/75)	93.3% (154/165)
NPV	NR	98.1%*
PPV	NR	77.5%*
Spared cystoscopies	NR	56.4%**

The number of conclusive patients, sensitivity, specificity, NPV, PPV and spared cystoscopies are shown for the discovery series (consisting of 26 bladder cancer patients, 18 prostate cancer patients, 12 renal cancer patients and 56 healthy controls) and the hematuria series (consisting of 273 patients with gross hematuria, including 93 patients with a confirmed bladder tumor)

*NR* Not relevant

*Calculated from sensitivity, specificity and a prevalence of 20%

**Calculated as the rate of true negatives among all patients in the series (undergoing cystoscopy with the current standard)

### BladMetrix performance among hematuria patients—a blinded analysis of a prospectively collected urine series

The cutoff to score a positive test, identified from the discovery series (Fig. 2; Additional file 1: Table S6), was quality controlled and validated in the independent prospective and blinded hematuria urine series by subsampling (*n* = 273; Additional file 1: Table S8). In the complete hematuria urine series, 93.0% of the patients (254/273) had a conclusive test result. Among those, BladMetrix achieved a sensitivity of 92.1% (82/89), a specificity of 93.3% (154/165), and a negative predictive value (NPV) of 98.1% for detection of bladder cancer (Table 1 and Additional file 1: Table S7). Finally, considering that BladMetrix identified 154 true negatives across the whole series of 273 patients, the test shows potential to reduce the number of cystoscopies by 56.4%.

### BladMetrix performance across tumor stages and grades

All low-stage and low-grade lesions were detected in the discovery urine series (100% sensitivity). In the hematuria series, Ta tumors and low-grade lesions were detected with a sensitivity of 84.6% and 85.2%, respectively (Table 2). All T2–T4 lesions were correctly scored in both urine series. False negative cases included one high-grade carcinoma in *situ* (CIS) in the discovery series, as well as 6 Ta tumors (4 low-grade and 2 high-grade) and 1 T1 tumor (high-grade) in the hematuria series.

**Table 2:** BladMetrix sensitivity across tumor stages and grades

	BlCa cases, discovery series (*n* = 26)	BlCa cases, hematuria series (*n* = 93)
Conclusive patients	88.5% (23/26)	95.7% (89/93)
*Stages*		
Ta	100% (11/11)	85% (33/39)
T1	–	97% (30/31)
T2	100% (3/3)	100% (9/9)
T3	100% (4/4)	100% (1/1)
T4	–	100% (2/2)
CIS	80% (4/5)	100% (7/7)
*Grade*		
Low-grade	100% (7/7)	85% (23/27)*
High-grade	94% (15/16)	95% (57/60)*

The sensitivity of BladMetrix to detect bladder tumors of different stages and grades is shown for the discovery series and the hematuria series

*BlCa* Bladder cancer

*Grade is missing for two of the included patients

## Discussion

We here present BladMetrix, a novel urine DNA methylation test for detection of bladder cancer with high diagnostic accuracy and potential to spare a significant number of cystoscopies. When analyzed in a large, blinded and prospective series of urine from patients with gross hematuria, BladMetrix achieved 92.1% sensitivity, 93.3% specificity and a NPV of 98.1%. Importantly, the sensitivity remained high across all tumor grades and stages, detecting Ta and low-grade tumors with around 85% sensitivity. In addition, BladMetrix showed potential to reduce the number of cystoscopies by 56.4% among gross hematuria patients.

Cystoscopy remains the gold standard for detection of bladder cancer, including in hematuria patients, but is a costly and invasive procedure that can be uncomfortable for the patients. In addition, the diagnostic accuracy is suboptimal. In several meta-analyses, the pooled sensitivity and specificity of the routinely used white light cystoscopy (WLC) has been estimated to around 70% [5, 18]. Variants of WLC include narrow band imaging (NBI) and photodynamic diagnosis (PDD), both methods shown to have higher sensitivity (96% and 93%, respectively), but lower specificity (65% and 63%, respectively) compared to WLC [18]. Screening of patients with an accurate molecular urine test such as BladMetrix, possibly followed by NBI or PDD, could represent a highly beneficial alternative to the current standard using WLC. Interestingly, it has been reported that the bladder cancer detection rate using cystoscopy improves if the urologist has been informed about a positive test result upfront [19]. A urine test stratifying for cystoscopy examination would be particularly useful among patients with gross hematuria, a patient group where the incidence rate of bladder cancer is as low as 17% [4], meaning that an unnecessary high number of cystoscopies are being performed.

Despite a handful of FDA-approved urinary tests and a variety of studies in the literature, accurate, robust and reliable biomarkers are still lacking, and no urine-based molecular tests are routinely used in the clinic [20]. Relevant criteria for a urine test with potential for clinical use in hematuria patients are improved accuracy compared to cystoscopy, high sensitivity across tumor grades, and high specificity considering other urological cancers. Moreover, the great majority of bladder cancer patients report that they are not willing to replace cystoscopy with a urinary test that has less than 90% sensitivity [21, 22]. These criteria are fulfilled by BladMetrix, including both sensitivity and specificity above 90% in a prospective series of hematuria patients, high sensitivity for low-grade tumors (85%) and high specificity against other urological cancers (95%). This level of accuracy across tumor stages and grades is rare among urinary tests for bladder cancer detection, and has to the best of our knowledge not been reached by any stand-alone commercially available urine test [11–13]. Of note, AssureMDX have been reported in the literature with 93–97% sensitivity for detection of bladder cancer in patients with gross hematuria, but has lower specificity (83–86%) compared to BladMetrix [23, 24]. Among non-commercial molecular tests, highly promising results have been published for UroMark, with 98% sensitivity and 97% specificity for detecting bladder cancer in hematuria patients, and clinical trials are ongoing to validate the results [25]. Also of note, Dahmcke et al. has reported a urine-DNA test with 97% sensitivity in the same prospective and blinded hematuria series as analyzed in this study, however with lower specificity (77%) [26].

Among bladder cancer patients, low-grade Ta tumors represent the majority of cases at diagnosis [9], and a test with high accuracy for these lesions is thus of great clinical interest. While cytology [5], several of the FDA-approved tests [8] and the majority of other published urinary biomarkers [12] typically have poor sensitivity for low-stage and low-grade tumors, BladMetrix show high accuracy also for these lesions with a detection rate of around 85% (Table 2). Importantly, this is comparable to WLC, which has been shown to miss up to 17% of early stage Ta tumors [27].

A limited number of hematuria urine samples (7%; 19/273) analyzed in the present study were scored inconclusive (i.e., 1/8 methylated biomarkers). The consequence for these patients in a clinical scenario would be to undergo cystoscopy (Fig. 2), which is in line with the current standard routine for hematuria patients. Importantly, despite a small percentage of inconclusive tests, we show that over half of the patients could have been spared an unnecessary cystoscopy using the BladMetrix test.

Our strategy for biomarker discovery is based on methylome sequencing of cancer cell lines. Cell lines have previously been shown to be highly suited for identification of accurate cancer biomarkers [17, 28, 29], which was confirmed in the present study, where the biomarker panel had high accuracy in both tissue and urine. As expected from a genome-wide approach, biomarkers were not restricted to areas of the genome with known biological function such as promoters. The majority of the candidates (59%) were located more than 1,500 bp away from the nearest TSS, indicating that intergenic regions are relevant sources for novel biomarkers.

The use of various material sources (cancer cell lines, tissue, urine), distinct clinical settings (patients scheduled for surgery *vs.* standard urological evaluation of hematuria patients), independent sample collection procedures in two countries and different methods for urine processing (centrifugation *vs.* filtration [30]) demonstrates the robustness of BladMetrix in various contexts. It also opens up for application in other clinical settings. A Norwegian multi-center trial is ongoing, aiming at following 500 patients with non-muscle-invasive bladder cancer over a two-year period to evaluate the clinical utility of BladMetrix for surveillance.

## Conclusions

In conclusion, we here present BladMetrix, a novel urine DNA methylation test for accurate detection of bladder cancer. In a large urine series from patients with gross hematuria, BladMetrix achieved high sensitivity and specificity across tumor stages and grades, and showed potential to spare over half of the cystoscopies that are routinely performed in this patient group. Given the high accuracy, cost efficiency, non-invasive nature and straightforward implementation, BladMetrix shows promise as a clinical test for urine-based detection of bladder cancer in hematuria patients.

## Methods

### Discovery of biomarker candidates from methylome sequencing

Sixteen urological cancer cell lines—eight bladder cancer, four prostate cancer and four renal cancer—were obtained from the American Type Culture Collection (ATCC). All cell lines were STR-tested and authenticated (Additional file 1: Table S9). RRBS library construction and sequencing were performed at Beijing Genomics Institute as previously described [31]. The data were processed according to the bioinformatics workflow described in Additional file 1: Methods, Fig. S1 and Table S10. DMRs were identified using a sliding window approach as illustrated in Additional file 1: Fig. S3.

### Identification of the most promising biomarker candidates in tissue

The biomarker candidates identified from RRBS were evaluated in 60 tissue samples, including 20 bladder cancer, 10 prostate cancer, 10 renal cancer and 20 normal bladder mucosa samples (Additional file 1: Materials). Biomarker candidates displaying > 50% sensitivity and having a significant AUC were considered for methylation analysis in urine using ddPCR, and the best performing biomarkers were combined into a panel, named BladMetrix.

### Analysis of the biomarker panel in urine – discovery and hematuria series

BladMetrix was first analyzed in the urine discovery series (*n* = 112; 26 bladder cancers, 18 prostate cancers, 12 renal cancers, 56 healthy controls). Different cutoffs for scoring a positive urine test were investigated, varying from ≥ 1/8 to 8/8 methylated biomarkers. The cutoff providing the most optimal combination of sensitivity, specificity, and number of conclusive samples was selected and applied for analyses in an independent urine series including 273 patients with gross hematuria, of which 93 had a clinically confirmed bladder cancer [26]. All patients considered for inclusion underwent TURB with biopsy and histological evaluation, and histological diagnosis was used as the standard reference.

Clinical data for the bladder cancer patients in both urine series (discovery- and hematuria series) is shown in Table 3. See Additional file 1: Materials and Table S11 for more information.

**Table 3:** Patient characteristics for the bladder cancer patients included in the discovery and hematuria urine series

	BlCa cases, discovery series (*n* = 26)	BlCa cases, hematuria series (*n* = 93)
*Gender*		
Male	65% (17/26)	78% (73/93)
Female	35% (9/26)	22% (20/93)
Median age, yr (range)	75 (49–93)	69 (48–91)
*Stage (n)*		
Ta	11	43
Ta + CIS	1	–
T1	–	22
T1 + CIS	–	9
T2	3	8
T2 + CIS	1	1
T3	2	1
T3 + CIS	2	–
T4	1	2
CIS	5	7
*Grade*		
Low-grade	31% (8/26)	31% (29/93)*
High-grade	69% (18/26)	66% (61/93)*

*BlCa* Bladder cancer; *yr* Year

*Grade is missing for three of the patients

### Urine processing, DNA isolation and bisulfite conversion

Urine samples in the discovery series were processed within 4 h using a standard centrifugation protocol, and DNA was isolated from urine pellets using the QIAamp DNA Mini Kit (Qiagen, Hilden, Germany). Urine samples in the hematuria series were processed using a urine filtration device, and DNA was purified from lysed cell samples using the Oragene DNA purifying solution (DNA Genotek, Ottawa, Canada) as previously described [30]. For all samples, the EpiTect Bisulfite Kit (Qiagen) was used for DNA bisulfite conversion. See Additional file 1: Methods for details.

### Targeted methylation analysis—qMSP and ddPCR

Bisulfite-treated DNA from tissue samples was analyzed by quantitative methylation-specific PCR (qMSP) using the 7900HT Real-Time PCR System (Life Technologies, Carlsbad, CA, USA) as previously described [32]. Urine DNA was analyzed by ddPCR using the QX200™ Droplet Digital™ PCR System (BioRad, Hercules, CA, USA) as previously described [33], and positive droplets were called using our PoDCall software [33] (https://bioconductor.org/packages/PoDCall/). See Additional file 1: Methods for details. All primer and probe sequences are listed in Additional file 1: Table S12. All analyses were performed according to the updated digital MIQE-guidelines (Additional file 1: Table S13) [34]. The “Standards for Reporting Diagnostic accuracy studies” (STARD) checklist [35] is included as Additional file 1: Table S14.

### Statistics

Statistical analyses were performed using IMB SPSS Statistics 25 and GraphPad Prism 9.1.2. Receiver operating characteristics (ROC) curve were used to evaluate the performance of the individual biomarker candidates. The area under the ROC curves (AUC), sensitivities, specificities and 95% confidence intervals were calculated considering conclusive test results [36]. The methylation concentrations providing the highest possible sensitivities with specificities > 95% (based on the discovery series: bladder cancer and healthy controls) were chosen as thresholds for scoring a sample as positive or negative for the individual biomarkers. Negative and positive predictive value (NPV and PPV) were calculated considering the sensitivity, specificity and a disease prevalence of 20% in hematuria patients [26].

## Supplementary Information

**Additional file 1.** Supplementary data.

## Data Availability

The methylome sequencing data are available from the corresponding author upon reasonable request. All other data that supports the findings of this study are available in the article and in the Additional file 1.

## Abbreviations

ATCC: American type culture collection
AUC: Area under the ROC curve
CIS: Carcinoma in situ
ddPCR: Droplet digital PCR
DMR: Differentially methylated region
FDA: The US food and drug administration
MIQE: Minimum information for publication of quantitative PCR experiments
NBI: Narrow band imaging
NPV: Negative predictive value
PDD: Photodynamic diagnosis
PPV: Positive predictive value
qMSP: Quantitative methylation-specific PCR
ROC: Receiver operating characteristics
RRBS: Reduced representation bisulfite sequencing
TSS: Transcription start site
WLC: White light cystoscopy
