# Supplementary material for: BladMetrix: a novel urine DNA methylation test with high accuracy for detection of bladder cancer in hematuria patients
Source: Clin Epigenetics. 2022 Sep 17;14:115. doi: 10.1186/s13148-022-01335-2 (PMC9482155; doi:10.1186/s13148-022-01335-2)
Supplement: Supplementary file 1 — Additional file 1. Supplementary data. [file 13148_2022_1335_MOESM1_ESM.docx]

SUPPLEMENTARY DATA

# Supplementary Materials

- 1. Clinical samples

**Tissue**

Bladder cancer tissue (n=20), renal cancer tissue (n=10) and normal bladder mucosa (n=20) was obtained from a consecutive series of patients diagnosed and treated at the Portuguese Oncology Institute of Porto, Porto, Portugal.

Prostate cancer tissue (n=10) was collected during prostatectomies undertaken at the Norwegian Radium Hospital, Oslo University Hospital, Oslo, Norway.

**Urine**

*Discovery series:*

Urine (3.5-180 ml) was collected from bladder cancer patients at the Aker University Hospital, Oslo University Hospital, Oslo, Norway, from January to May 2017, as well as from prostate cancer patients and renal cancer patients treated at the same hospital. The bladder cancer cohort consisted of patients scheduled for TURB (either first visit or under follow-up for recurrence) or cystectomy. Only patients with a histologically confirmed cancer were included.

Urine (24-150 ml) was also obtained from self-declared healthy volunteers working at the Norwegian Radium Hospital (collected in May 2017) or being a part of a voluntary aid organization (collected in April 2021). The normal urine sample cohort consisted of 32% men with a median age of 58.5 years.

*Hematuria series:*

Urine was collected prospectively from consecutive patients undergoing standard urological evaluation for gross hematuria, including flexible cystoscopy, urinary cytology, and computed tomography urography, in the outpatient clinic at Herlev Hospital, between December 2013 and January 2015 as previously described^1^.

For both urine series, the included patients and controls were asked to provide two parallel urine samples, *i.e.* from two independent urinations, before and/or after the clinical intervention (TURB, cystoscopy or cystectomy; Supplementary Table 11).

# Supplementary Methods

2.1 Bioinformatic identification of biomarker candidates from RRBS data

The workflow established for identification of DMRs from raw RRBS data is shown in Supplementary Figure 1. Quality control of the sequence reads was performed using FASTQC (version 0.10.1). Low quality reads and Illumina adapters (Supplementary Table 15) were trimmed using the wrapper script TrimGalore (version 0.3.3), which uses Cutadapt and checks quality after trimming using FASTQC. Trimmed reads were aligned to a bisulfite converted version of the human reference genome (hg19) using Bismark (version 0.10.0), and ran with Bowtie2. Of note, all samples showed near complete bisulfite conversion (≥99.5%; Supplementary Table 16). Samtools (version 0.1.18) was employed to edit the alignment files. MethylKit (version 0.9.2) was used to calculate the methylation status of each CpG site covered.

DMRs were identified across the different urological cancer types using a sliding window approach. Windows of 200 bp and 1000 bp, using a 100-bp step size between windows, were utilized. A SQL database (SQLite studio version 3.0.5) storing the lists of DMRs was created, and specific queries were applied to filter out relevant regions. Regions with a methylation difference of >25% between bladder cancer and prostate/renal cancer, as well as ≥50% methylation in at least 7/8 of the bladder cancer cell lines and <20% methylation in at least 6/8 of the prostate/renal cancer cell lines, were considered as biomarker candidates. Bedgraph files from BAM files (Bismark methylation extractor) were used for visualization of regions of interest in Integrative Genomics Viewer (IGV, version 2.3).

2.2 Urine filtration, DNA isolation and bisulfite conversion

For the hematuria urine series, voided urine samples (100–400 ml) were processed using a filtration device mounted with a Nuclepore track-etched polycarbonate hydrophilic membrane filter (diameter 25 mm, pore size 8 μm; Whatman, Maidstone, UK). After filtration, the filter cartridge was transferred to the storage cassette, which was then mounted with the lid from an Oragene DNA Self-Collection Kit containing 1.7 ml of lysis/stabilization buffer (disc format OG-250; DNA Genotek, Ottowa, Ontario, Canada). According to the manufacturer, DNA is stable in this solution for >5 years at room temperature (modified from Andersson et al. PLoS One. 2015^2^).

DNA was isolated from urine pellets using the QIAamp DNA Mini Kit (Qiagen, Hilden, Germany) for the urine samples in the discovery series, and as previously described for urine in the hematuria series^1^. DNA from tissue and cell lines were isolated as previously described^3,4^.

For all samples, the EpiTect Bisulfite Kit (Qiagen) was used for bisulfite conversion of DNA. Bisulfite converted samples were automatically purified by the QIAcube System (Qiagen) and eluted in 40 µl EB-buffer (Qiagen). All kits were used according to the manufacturers’ standard protocols. The input amount in the bisulfite conversion reaction was 1300 ng for cancer cell lines and tissue and 430 ng for urine in the discovery series, and all samples were diluted to ~10ng/µl after purification and elution. For the large and independent hematuria urine series, 20 µl was used as input in the bisulfite conversion. Of note, a pilot of random samples from the discovery urine series was run up front (data not shown) to ensure concordant data between a DNA amount based input approach (cell lines, tissue, discovery urine series) and a DNA volume based input approach (hematuria urine series), and as expected, the 4Plex control included in the ddPCR analyses ensured robust normalized data (see 2.3 “Targeted DNA methylation analyses: quantitative methylation-specific PCR (qMSP) and droplet digital PCR (ddPCR)” below). No bladder cancer patients were excluded due to insufficient DNA amounts (discovery series ≥430 ng; hematuria series: no limit), but for two of the patients in the discovery series, one of the two urinates was excluded due to too low DNA concentration (<10 ng/ul). In addition, one patient was excluded due to low 260/280 and 230/260 ratios (as measured on the NanoDrop 1000 Spectrophotometer; Thermo Fisher Scientific)).

2.3 Targeted DNA methylation analyses: quantitative methylation-specific PCR (qMSP) and droplet digital PCR (ddPCR)

**Quantitative methylation-specific PCR (qMSP)** of 32.5 ng bisulfite-treated DNA from tissue samples was performed using the 7900HT Real-Time PCR System (Life Technologies, Carlsbad, CA, USA). The repetitive element ALU-C4^5^ was used for normalization. The percentage of methylated molecules per sample (PMR value) was calculated by dividing the normalized quantity (target/ALU-C4) of the samples by the normalized quantity of the methylation positive control (universal methylated human DNA standard; Zymo Research, Irvine, CA, USA), and multiplying by 100. The highest PMR value in normal bladder mucosa was used as threshold for positive methylation scoring, ensuring assay specificity.

**Droplet digital PCR (ddPCR)** was performed using the QX200™ Droplet Digital™ PCR System (BioRad, Hercules, CA, USA). The ddPCR reaction consisted of 1x ddPCR Supermix for Probes (BioRad), 818 nM of each primer, 182 nM of each probe and 3 µl bisulfite converted DNA template in a final volume of 22 µl. Droplet generation of 20 µl of the pre-reaction volume was performed using either the QX200 Droplet Generator (BioRad), or the Automated Droplet Generator (BioRad). The PCR was performed either in a T100 Thermal Cycler (BioRad) or in a Tetrad 2 Thermal Cycler (BioRad). See Supplementary Table 17 for PCR cycling conditions.

Positive droplet calling was performed using the PoDCall algorithm (<https://bioconductor.org/packages/PoDCall/>) and the 4Plex control^6^ was used for normalization. A minimum of 3 positive droplets was required for methylation scoring of the target gene, *i.e.* the biomarkers. The ddPCR methylation concentrations (cop/µl) were calculated by dividing the concentration of the target by the concentration of the 4Plex, and multiplying by a constant of 400. For the discovery series (collected with a standard centrifugation protocol resulting in generally high DNA yields) a 4Plex lower limit of 100 cop/µl was applied, while for the hematuria series (collected with the filtration device enriching for tumor cells^2^), the 4Plex lower limit was set to 1.5 cop/µl, based on the highest signal observed in the non-template controls (NTCs). In the discovery series, no bladder cancer patient was excluded due to too low 4plex (<100 cop/µl), but for three of the patients, one of the two parallel urine samples was excluded. For the hematuria series, 31 samples from 19 patients displayed a 4Plex under the 1.5 cop/µl limit and were excluded from further analyses.

The samples were scored as positive or negative for methylation of the individual biomarkers based on thresholds obtained from ROC curve analyses of the discovery series (cancer and healthy controls) - the methylation concentrations providing the highest possible sensitivities with specificities >95% was chosen as thresholds (Supplementary Table 18). For patients with two parallel urinates, the highest methylation concentration per biomarker was used. For all analyses, universal methylated human DNA standard (Zymo Research) was used as methylation-positive control, normal blood of healthy donors and/or human WGA non-methylated DNA (Zymo Research) as methylation-negative controls, and RNase-free water (Sigma Aldrich, St. Louis, Missouri, USA) as non-template control (NTC). See Supplementary Figure 6 for a representative example of amplification of positive and negative controls.

**References for Supplementary Materials and Methods**

1. Dahmcke CM, Steven KE, Larsen LK, et al. A Prospective Blinded Evaluation of Urine-DNA Testing for Detection of Urothelial Bladder Carcinoma in Patients with Gross Hematuria. *Eur Urol.* 2016;70(6):916-919.
2. Andersson E, Dahmcke CM, Steven K, Larsen LK, Guldberg P. Filtration Device for On-Site Collection, Storage and Shipment of Cells from Urine and Its Application to DNA-Based Detection of Bladder Cancer. *PLoS One.* 2015;10(7):e0131889.
3. Costa VL, Henrique R, Danielsen SA, et al. Three epigenetic biomarkers, GDF15, TMEFF2, and VIM, accurately predict bladder cancer from DNA-based analyses of urine samples. *Clin Cancer Res.* 2010;16(23):5842-5851.
4. Lovf M, Zhao S, Axcrona U, et al. Multifocal Primary Prostate Cancer Exhibits High Degree of Genomic Heterogeneity. *Eur Urol.* 2018.
5. Weisenberger DJ, Campan M, Long TI, et al. Analysis of repetitive element DNA methylation by MethyLight. *Nucleic Acids Res.* 2005;33(21):6823-6836
6. Pharo HD, Andresen K, Berg KCG, Lothe RA, Jeanmougin M, Lind GE. A robust internal control for high-precision DNA methylation analyses by droplet digital PCR. *Clin Epigenetics*. 2018;10:24.

# Supplementary Figures

**
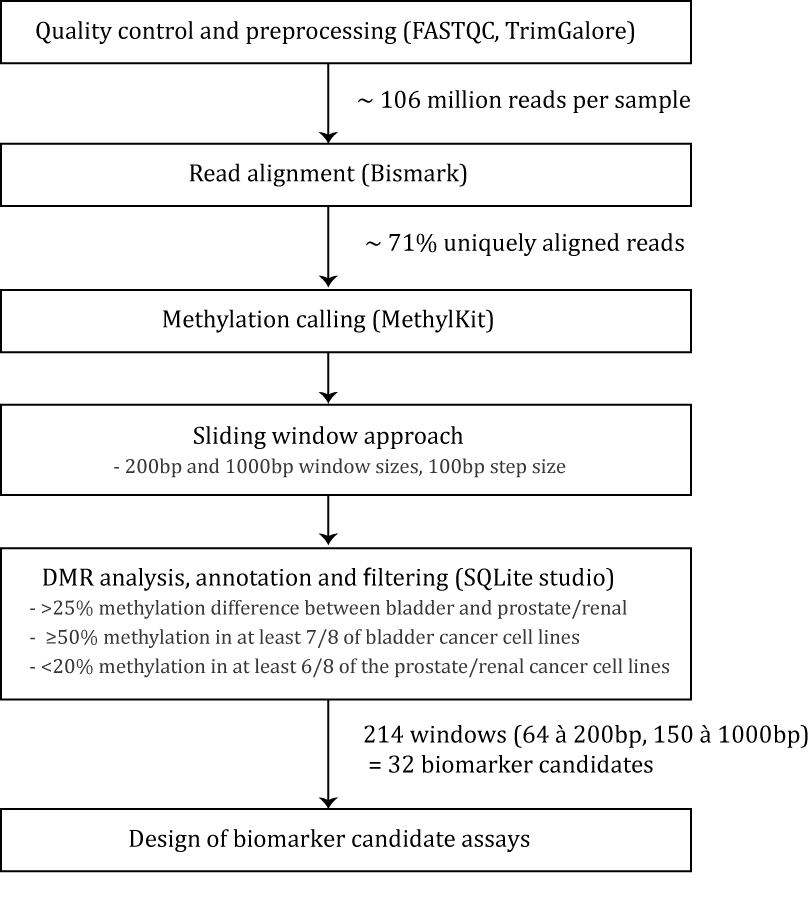
**

Supplementary Figure 1: Flow-chart of the in-house developed bioinformatic workflow for DNA methylation biomarker discovery. The workflow is explained in detail in Supplementary Methods. Abbreviations: DMR, differentially methylated region.


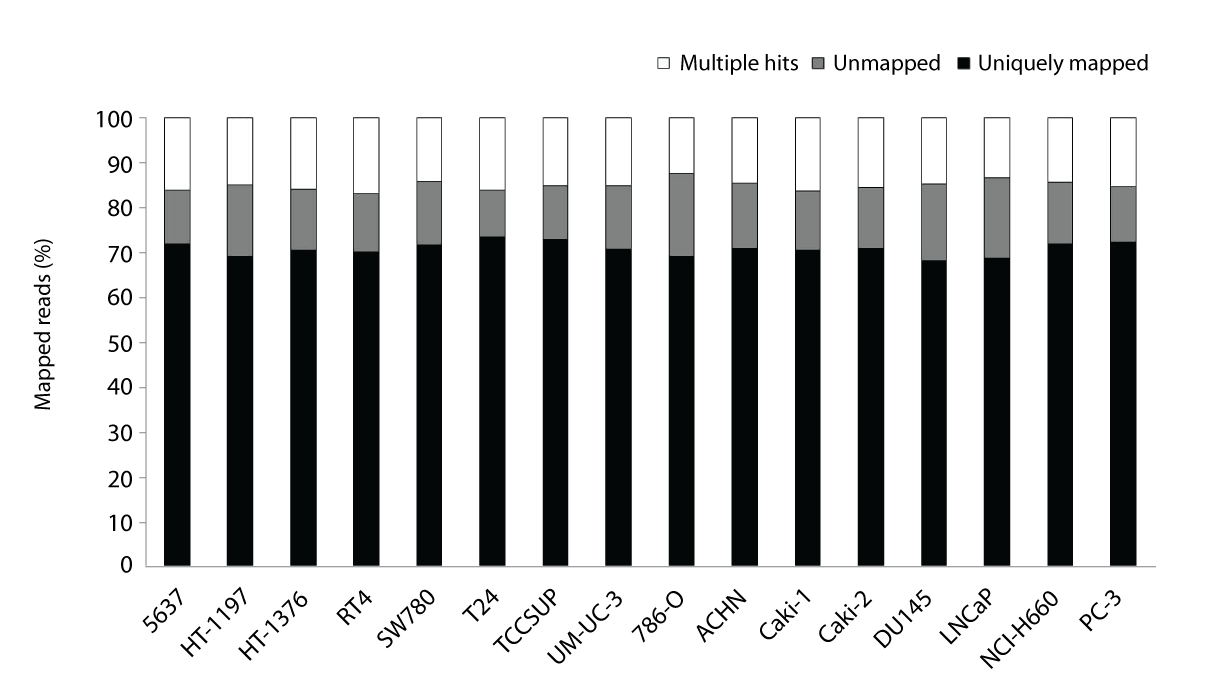
Supplementary Figure 2: Reduced representation bisulfite sequencing (RRBS) mapping efficiency of urological cancer cell lines. On average, 71% of the quality controlled and trimmed reads were uniquely mapped to the bisulfite treated genome (hg19). Moreover, 15% of the reads had multiple hits, and 14% were unmapped reads. Only uniquely mapped reads were kept for further analysis.

**
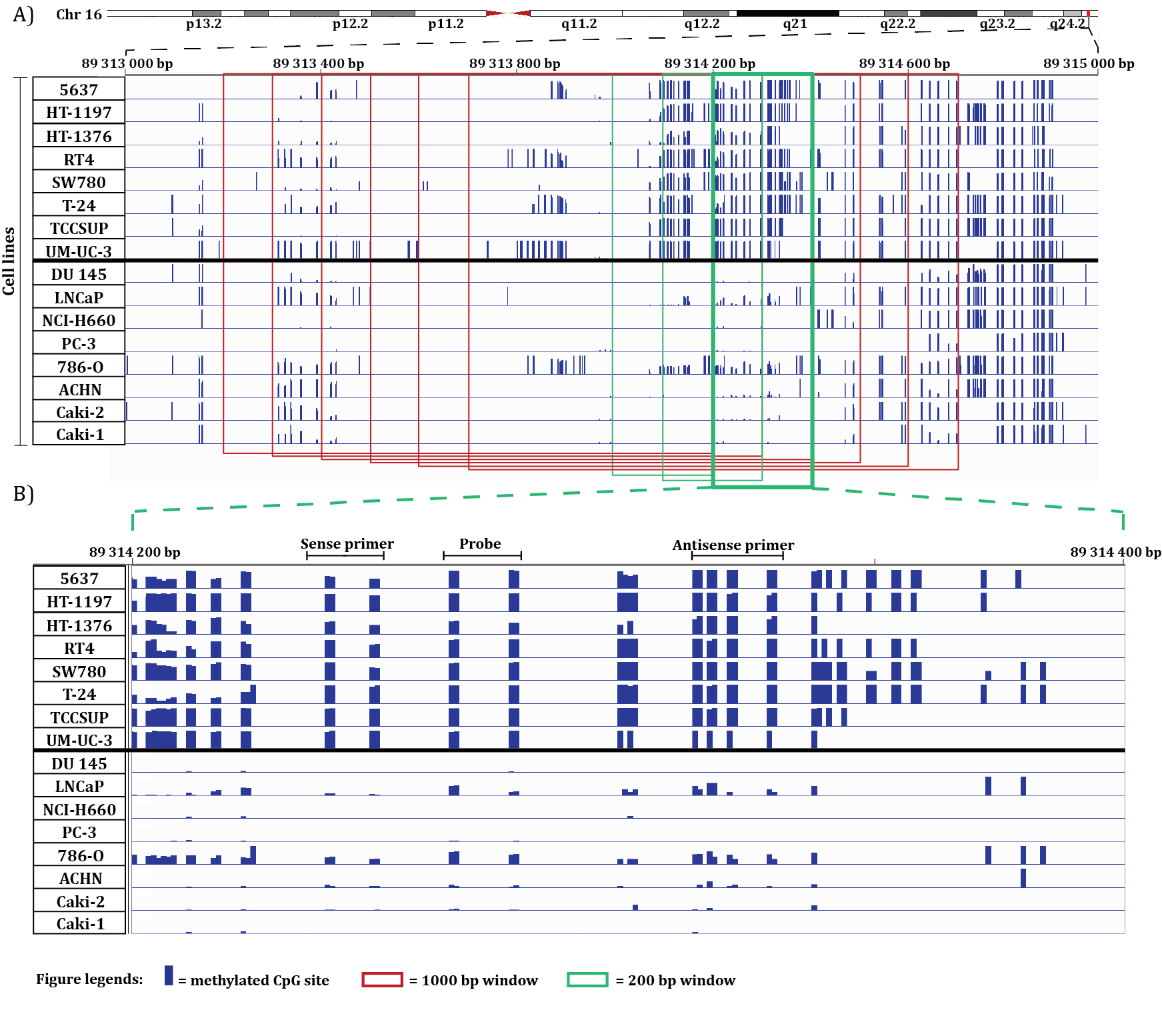
**

Supplementary Figure 3: A bladder cancer biomarker candidate identified from methylome sequencing. A) A representative biomarker candidate on chromosome 16 (16-89314201) is shown, including both 1000-bp windows (red boxes; n=6) and 200bp windows (green boxes; n=3) with 100-bp step size. The horizontal lines represent one cancer cell line, with the bladder cancer cell lines on top (n=8) and other urological cell lines below (n=8; separated with a dark bold line). Vertical blue bars indicate a methylated CpG site, and the height of the bars reflects the degree of methylation. B) A more detailed view of a relevant 200-bp window from A), including primer and probe locations for biomarker 16-89314201.


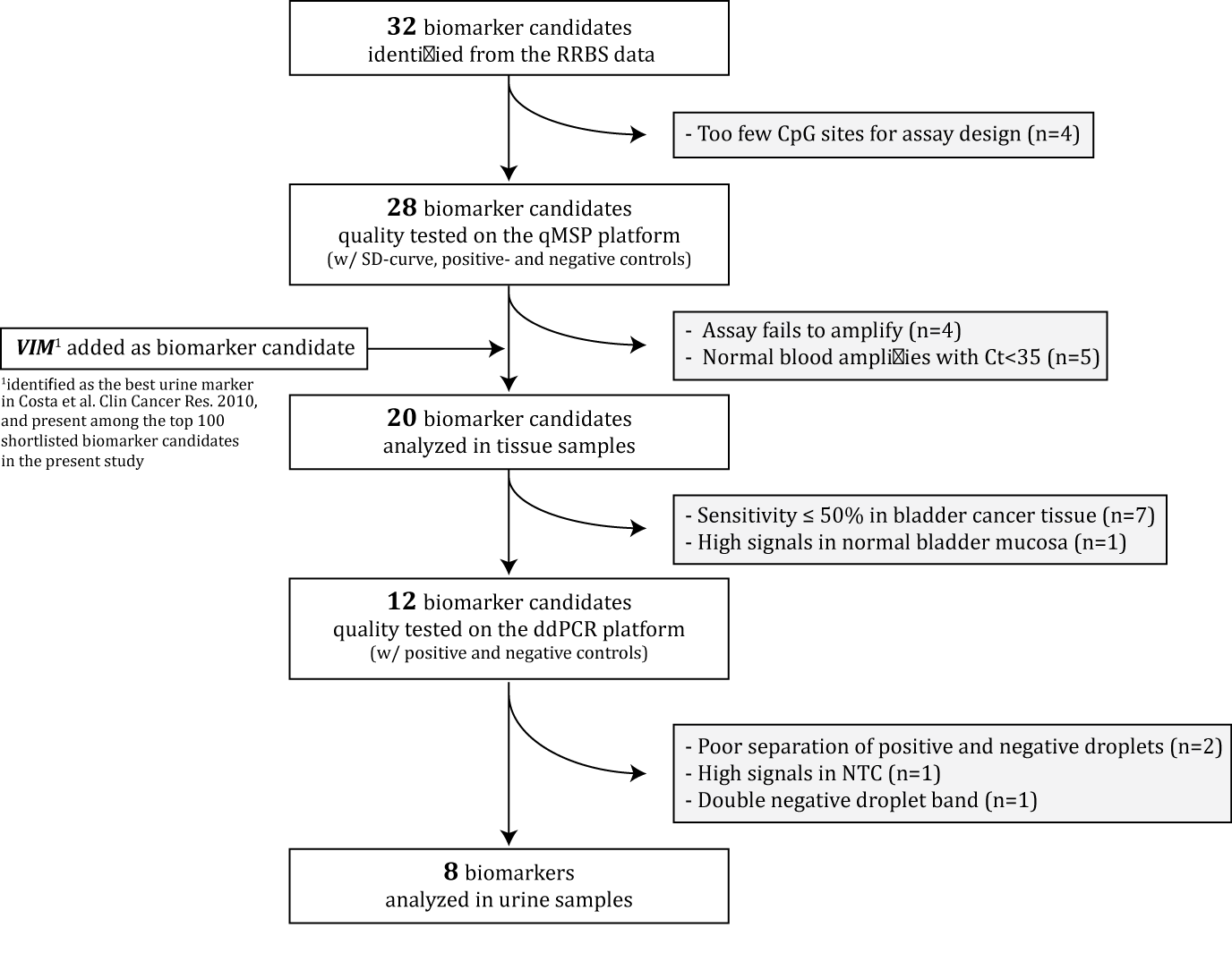


Supplementary Figure 4: Stepwise selection of DNA methylation biomarkers based on an initial list of 32 candidates identified from methylome sequencing. Abbreviations: Ct, threshold cycle; ddPCR; droplet digital PCR; NTC, non-template control; qMSP, quantitative methylation-specific PCR; RRBS, reduced representation bisulfite sequencing; SD-curve, standard curve.


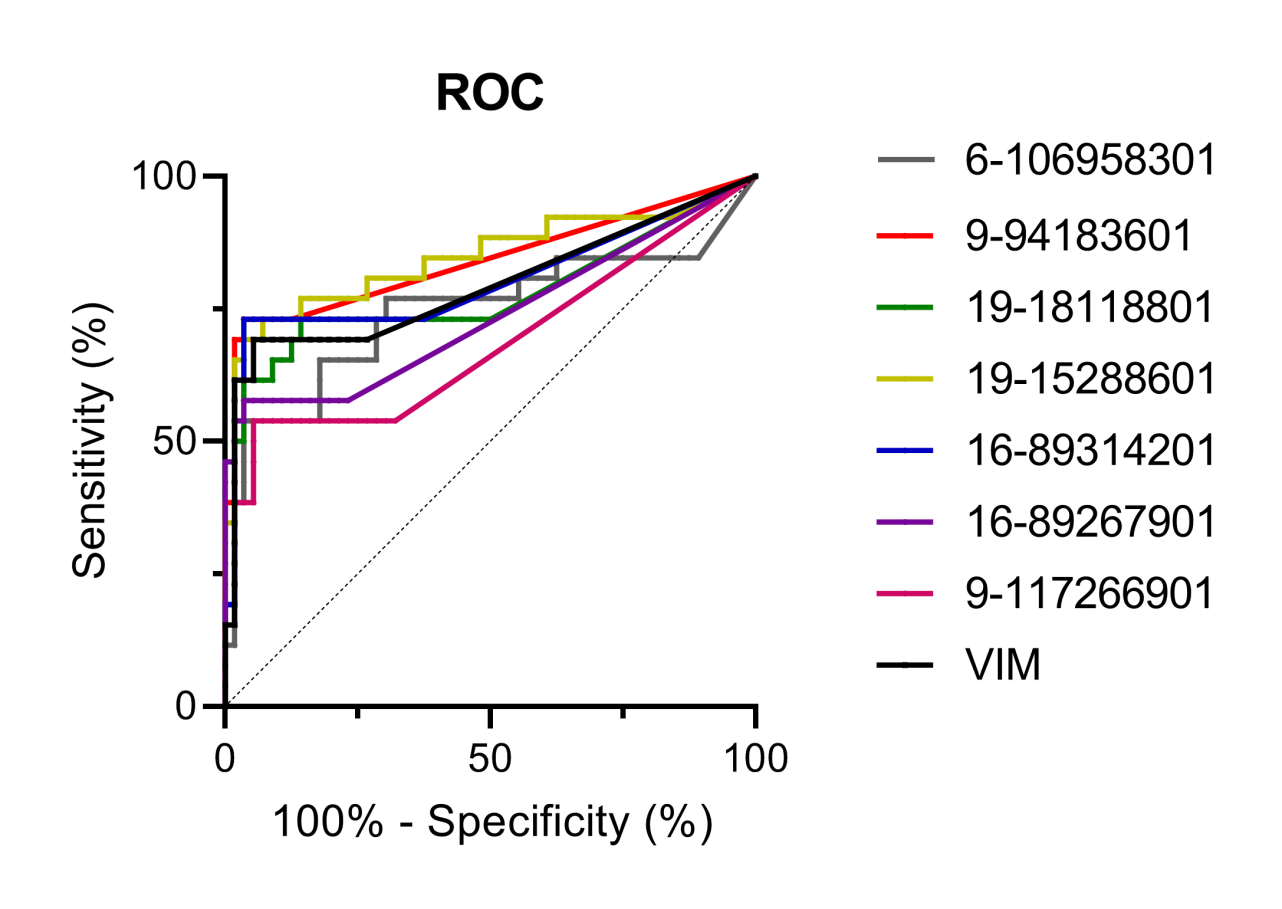


**Supplementary Figure 5: Receiver operator characteristics (ROC) curves for the DNA methylation biomarkers in the urine discovery series.** Individual biomarker thresholds are computed based on their ability to discriminate the 26 bladder cancer patients *vs.* the 56 healthy controls.

**
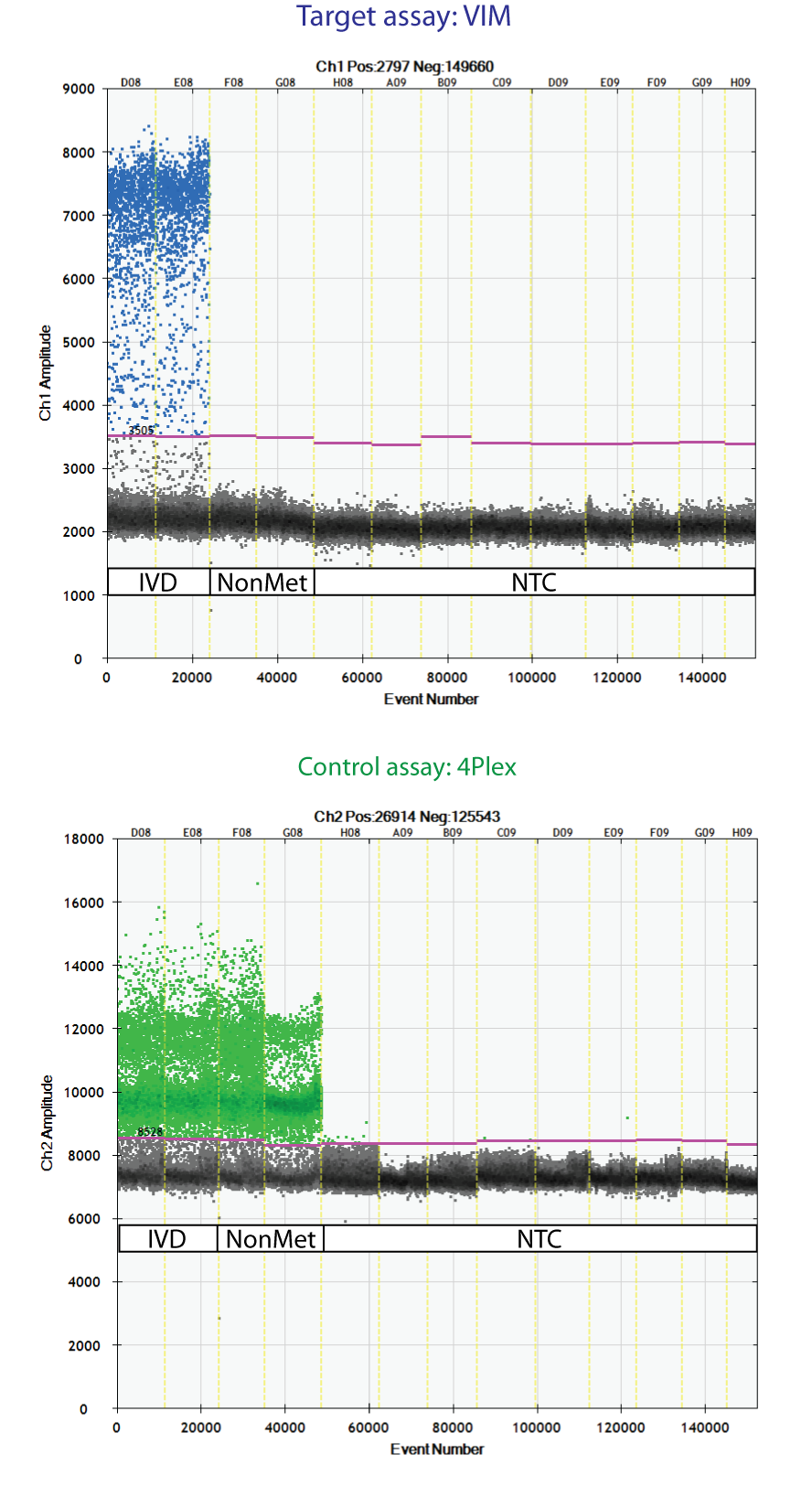
**

Supplementary Figure 6: A representative example of ddPCR amplification of positive and negative controls. The target assay (*VIM*) is shown in in the upper plots, and the control assay (4Plex) in the lower plots. The samples are (from left to right); two methylation positive-controls (IVD; *i.e.* universal methylated human DNA standard; Zymo Research), two methylation-negative controls (NonMet; *i.e.* human WGA non-methylated DNA; Zymo Research), and 9 non-template controls (NTC; *i.e.* RNase-free water; Sigma Aldrich). The horizontal pink lines represent the thresholds determined by PoDCall to separate the positive droplets (blue for VIM, green for 4Plex) and negative droplets (grey). Abbreviations: IVD, *in vitro* methylated DNA; NonMet, non-methylated DNA; NTC, non-template control.

# Supplementary Tables

|  |  |  |
| --- | --- | --- |

|  | Bladder cancer cell lines | | | | | | | | Prostate cancer cell lines | | | | Renal cancer cell lines | | | | Mean |
| --- | --- | --- | --- | --- | --- | --- | --- | --- | --- | --- | --- | --- | --- | --- | --- | --- | --- |
|  | 5637 | HT-1197 | HT-1376 | RT4 | SW780 | T-24 | TCCSUP | UM-UC-3 | DU 145 | LNCaP | NCI-H660 | PC-3 | 786-O | ACHN | Caki-1 | Caki-2 |  |
| Total number of reads | 1.06E+08 | 1.06E+08 | 1.06E+08 | 1.06E+08 | 1.06E+08 | 1.06E+08 | 1.06E+08 | 1.06E+08 | 1.06E+08 | 1.06E+08 | 1.06E+08 | 1.06E+08 | 1.06E+08 | 1.06E+08 | 1.06E+08 | 1.06E+08 | 1.06E+08 |
| Uniquely mapped reads (%) | 71.9 | 69.2 | 70.5 | 70.2 | 71.7 | 73.5 | 73.0 | 70.8 | 68.3 | 68.7 | 72.0 | 72.3 | 69.3 | 71.0 | 70.5 | 71.0 | 70.9 |
| Average coverage (x) | 100.0 | 126.6 | 126.6 | 100.5 | 101.7 | 105.6 | 108.8 | 111.8 | 125.8 | 109.2 | 119.8 | 107.6 | 96.9 | 106.9 | 119.4 | 118.8 | 111.6 |
| Average methylation (%) | 37.6 | 29.0 | 22.4 | 43.3 | 32.0 | 37.3 | 29.8 | 46.2 | 41.2 | 34.9 | 37.3 | 37.8 | 39.6 | 42.3 | 38.5 | 46.6 | 37.2 |

**Supplementary Table 1: Results from the quality control of the RRBS raw data.** The table shows the total number of reads resulting after quality control and trimming, the mapping efficiency, the coverage, and the methylation distribution. All results are shown for individual cell lines, as well as on average across all cell lines.

| Marker no. | Marker name | Chr | Start pos window | End pos window | Distance to nearest gene TSS | Nearest gene | Gene ID |
| --- | --- | --- | --- | --- | --- | --- | --- |
| 1 | 1-26663001 | chr1 | 26663001 | 26663200 | 17422 | AIM1L | NM_001039775 |
| 2 | 1-234040601 | chr1 | 234040601 | 234040800 | 0 | SLC35F3 | NM_173508 |
| 3 | 2-1747901 | chr2 | 1747901 | 1748100 | 192 | PXDN | NM_012293 |
| 4 | 3-197808301 | chr3 | 197808301 | 197808500 | -760 | ANKRD18DP | NR_003291 |
| 5 | 6-106958301 | chr6 | 106958301 | 106958500 | -1230 | AIM1 | NM_001624 |
| 6 | 7-20824801 | chr7 | 20824801 | 20825000 | 1509 | SP8 | NM_198956 |
| 7 | 7-65878301 | chr7 | 65878301 | 65879300 | -12907 | LINC00174 | NR_026873 |
| 8 | 8-97173201 | chr8 | 97173201 | 97173400 | -182 | GDF6 | NM_001001557 |
| 9 | 9-34665101 | chr9 | 34665101 | 34665300 | -2413 | CCL27 | NM_006664 |
| 10 | 9-94183601 | chr9 | 94183601 | 94184600 | 1545 | NFIL3 | NM_005384 |
| 11 | 9-117266901 | chr9 | 117266901 | 117267100 | 637 | DFNB31 | NM_001173425 |
| 12 | 10-102810001 | chr10 | 102810001 | 102810200 | -10799 | KAZALD1 | NM_030929 |
| 13 | 10-133796001 | chr10 | 133796001 | 133796200 | -567 | BNIP3 | NM_004052 |
| 14 | 12-10306201 | chr12 | 10306201 | 10306400 | -16798 | TMEM52B | NM_001079815 |
| 15 | 12-49690901 | chr12 | 49690901 | 49691100 | 1994 | PRPH | NM_006262 |
| 16 | 12-54764201 | chr12 | 54764201 | 54764400 | -5932 | GPR84 | NM_020370 |
| 17 | 12-124854101 | chr12 | 124854101 | 124854300 | -32314 | MIR6880 | NR_106940 |
| 18 | 12-125534001 | chr12 | 125534001 | 125534200 | -15725 | AACS | NM_023928 |
| 19 | 13-45945201 | chr13 | 45945201 | 45945400 | 29723 | TPT1-AS1 | NR_024458 |
| 20 | 15-26108101 | chr15 | 26108101 | 26108300 | 50 | ATP10A | NM_024490 |
| 21 | 15-91498801 | chr15 | 91498801 | 91499800 | 697 | RCCD1 | NM_001017919 |
| 22 | 16-676701 | chr16 | 676701 | 677700 | -3312 | WFIKKN1 | NM_053284 |
| 23 | 16-67188901 | chr16 | 67188901 | 67189900 | 3913 | TRADD | NM_003789 |
| 24 | 16-88600201 | chr16 | 88600201 | 88600400 | -36389 | ZC3H18 | NM_144604 |
| 25 | 16-89267901 | chr16 | 89267901 | 89268100 | -1373 | SLC22A31 | NM_001242757 |
| 26 | 16-89314201 | chr16 | 89314201 | 89314400 | 30092 | ZNF778 | NR_037705 |
| 27 | 17-8055601 | chr17 | 8055601 | 8055800 | 0 | PER1 | NM_002616 |
| 28 | 17-38347601 | chr17 | 38347601 | 38347800 | -2049 | MIR6867 | NR_106927 |
| 29 | 17-40700201 | chr17 | 40700201 | 40701200 | -2784 | HSD17B1 | NM_000413 |
| 30 | 19-15288601 | chr19 | 15288601 | 15288800 | 1362 | MIR6795 | NR_106853 |
| 31 | 19-18118801 | chr19 | 18118801 | 18119000 | 0 | ARRDC2 | NM_001286826 |
| 32 | 22-27152801 | chr22 | 27152801 | 27153000 | 83997 | MIATNB | NR_110543 |

**Supplementary Table 2: List of the 32 biomarker candidates identified from RRBS data.**

| Assay name | Sensitivity (%) | Specificity  (%; NBM) | Specificity  (%; NBM, ReCa, PrCa) | AUC | p-value | 95%CI lower bound | 95%CI upper bound |
| --- | --- | --- | --- | --- | --- | --- | --- |
| 6-106958301 | 100 | 100 | 100 | 1,000 | <0,0001 | 1,000 | 1,000 |
| VIM | 95 | 100 | NA | 0,975 | <0,0001 | 0,920 | 1,000 |
| 9-94183601 | 90 | 100 | 92.5 | 0,969 | <0,0001 | 0,910 | 1,000 |
| 16-89314201 | 80 | 100 | 97.5 | 0,880 | <0,0001 | 0,760 | 1,000 |
| 17-38347601^1^ | 80 | 100 | NA | 0,904 | 2,00E-04 | 0,800 | 1,000 |
| 19-15288601^2^ | 75 | 95 | 97.5 | 0,846 | 2,00E-04 | 0,720 | 0,980 |
| 19-18118801 | 75 | 100 | 100 | 0,863 | <0,0001 | 0,730 | 0,990 |
| 3-197808301 | 75 | 100 | 97.5 | 0,850 | 2,00E-04 | 0,720 | 0,980 |
| 9-117266901 | 70 | 100 | 90 | 0,843 | 2,00E-04 | 0,710 | 0,970 |
| 10-133796001 | 70 | 100 | NA | 0,850 | 1,10E-03 | 0,720 | 0,980 |
| 16-89267901 | 65 | 100 | 92.5 | 0,808 | 9,00E-04 | 0,660 | 0,950 |
| 16-67189101 | 65 | 100 | NA | 0,825 | 2,40E-03 | 0,680 | 0,970 |
| 17-8055601 | 65 | 100 | NA | 0,781 | 8,60E-03 | 0,620 | 0,940 |
| 12-49690901 | 60 | 92 | NA | 0,602 | 3,40E-01 | 0,400 | 0,810 |
| 10-79397801 | 50 | 100 | NA | 0,750 | 1,95E-02 | 0,580 | 0,920 |
| 7-65878301 | 50 | 100 | NA | 0,542 | 6,97E-01 | 0,330 | 0,750 |
| 15-26108101 | 45 | 100 | NA | 0,725 | 3,56E-02 | 0,550 | 0,900 |
| 10-102810001 | 40 | 100 | NA | 0,700 | 6,17E-02 | 0,520 | 0,880 |
| 1-26663001 | 35 | 100 | NA | 0,675 | 1,02E-01 | 0,490 | 0,860 |
| 9-34665101 | 25 | 100 | NA | 0,625 | 2,43E-01 | 0,430 | 0,820 |

**Supplementary Table 3:** **Performance of DNA methylation biomarker candidates in tissue DNA.** The 20 assays are sorted by decreasing sensitivity in bladder cancer tissue (n=20). Only the most promising candidates were analyzed in tissue from prostate cancer (n=10) and renal cancer patients (n=10) due to limited amount of material. For the same reason, 20 normal bladder mucosa samples were included for analysis of these candidates, compared to 12 normal bladder mucosa samples for the remaining assays. Assays with sensitivity < 50% and/or non-significant AUC are highlighted in grey. AUC, *p*-value and 95% CI have been calculated considering normal bladder mucosa samples as controls (n=12). ^1^Assay excluded from further analyses due to high signals in normal bladder mucosa. ^2^For this assay, one normal bladder mucosa sample was scored positive due to high signal, resulting in a specificity of 95%. Abbreviations: AUC, area under the ROC-curve; BlCa, bladder cancer; CI, confidence interval; NA, not applicable; NBM, normal bladder mucosa; PrCa, prostate cancer; ReCa, renal cancer.

| Assay name | AUC | Sensitivity (%) | Specificity  (%; HC) | | Specificity  (%; HC, ReCa, PrCa) |
| --- | --- | --- | --- | --- | --- |
| 19-15288601 | 0.848 | 69 | | 96 | 94 |
| 9-94183601 | 0.842 | 73 | | 96 | 98 |
| 16-89314201 | 0.803 | 73 | | 96 | 95 |
| VIM | 0.793 | 69 | | 95 | 94 |
| 19-18118801 | 0.775 | 62 | | 96 | 97 |
| 6-106958301 | 0.745 | 54 | | 96 | 94 |
| 16-89267901 | 0.737 | 58 | | 96 | 98 |
| 9-117266901 | 0.683 | 54 | | 95 | 95 |

**Supplementary Table 4:** **Individual DNA methylation biomarker performance in the discovery series.** AUC, sensitivity and specificity for the eight most promising biomarkers are shown for the discovery series, consisting of 26 bladder cancers, 56 healthy controls, 18 prostate cancers and 12 renal cancers. The biomarkers are sorted by decreasing AUCs, which have been calculated using the healthy individuals as controls. Abbreviations: AUC, area under the ROC curve; BlCa, bladder cancer; HC, healthy control; PrCa, prostate cancer; ReCa, renal cancer.

|  | **6-106958301** | **9-94183601** | **19-18118801** | **19-15288601** | **16-89314201** | **16-89267901** | **9-117266901** | **VIM** |
| --- | --- | --- | --- | --- | --- | --- | --- | --- |
| **p-value** | 1.00 | 0.14 | 1.00 | 1.00 | 1.00 | 1.00 | 0.87 | 1.00 |

**Supplementary Table 5: Testing for potential age dependent methylation.** The p-values resulting from testing for potential age dependent methylation in the 26 bladder cancer cases in the discovery series when stratifying in two equally sized age groups (“old” and “young”) shows no significant association for any of the eight biomarkers (Wilcoxon + FDR correction, method = BH). The median age of the “old” and “young” age groups is 82 and 64 years, respectively.

| **Cutoff^*^** | **≥1/8** | **≥2/8** | **≥3/8** | **≥4/8** | **≥5/8** | **≥6/8** | **≥7/8** | **8/8** |
| --- | --- | --- | --- | --- | --- | --- | --- | --- |
| **Sensitivity (%)** | 96 | 96 | 95 | 94 | 94 | 94 | 92 | 89 |
| **Specificity (%)** | 83 | 95 | 96 | 96 | 97 | 99 | 99 | 99 |
| **Conclusive samples (%)** | 100 | 88 | 84 | 82 | 80 | 79 | 75 | 72 |

^*^The cutoff defines the number of methylated biomarkers required to score a positive test.

**Supplementary Table 6:** **The performance of BladMetrix in the urine discovery series using different cutoffs.** The performance of BladMetrix is displayed according to different cutoffs, meaning the number of methylated biomarkers used to score a sample as bladder cancer, *i.e.* a positive urine test. The cutoffs range from ≥1/8 to 8/8 methylated biomarkers. Prostate and renal cancer tissue samples were included in the control group to calculate specificity. Using a cutoff of ≥1/8 methylated biomarkers, the sensitivity reached 96% and the specificity was 83%; all samples were scored conclusive. For all other cutoffs (≥2/8 to 8/8), samples with a number of methylated biomarkers above 0, but below the cutoff, were considered inconclusive. The ≥2/8 methylated biomarkers cutoff (grey shading) resulted in the most optimal combination of sensitivity (96%), specificity (95%) and rate of conclusive samples (88%), and were selected for downstream analyses.

|  | Disease status | | | |
| --- | --- | --- | --- | --- |
| Test result | | **Bladder cancer** | **No bladder cancer** |  |
| Urine discovery series | | | |  |
| Positive | | 22 | 4 |  |
| Negative | | 1 | 71 |  |
| Inconclusive | | 3 | 11 |  |
| Urine hematuria series | | | |  |
| Positive | | 82 | 11 |  |
| Negative | | 7 | 154 |  |
| Inconclusive | | 4 | 15 |  |

**Supplementary Table 7: Classification table showing positive, negative and inconclusive BladMetrix test results in the urine discovery and hematuria series.**

|  | **Subset 1**  **(n=204; 75%)** | **Subset 2**  **(n=69; 25%)** |
| --- | --- | --- |
| **Conclusive tests (%)** | 93 | 94 |
| **Sensitivity (%)** | 90 | 96 |
| **Specificity (%)** | 92 | 97 |
| **NPV (%)** | 97 | 99 |
| **PPV (%)** | 74 | 90 |

**Supplementary Table 8: Subsampling of the hematuria urine series to validate the cutoff for scoring of a positive test.** The cutoff of ≥2/8 methylated biomarkers to score a positive BladMetrix test (identified from the discovery urine series) was quality controlled and validated in the hematuria urine series by random splitting of the series into subset 1 (75% of the samples) and subset 2 (25% of the samples). The percentage of conclusive tests, sensitivity, specificity, NPV and PPV in the two subsets are shown. Abbreviations: NPV, negative predictive value; PPV, positive predictive value.

| Cancer cell line | Tissue | Supplier | Supplier ID | Supplier ID confirmed by STR profiling |
| --- | --- | --- | --- | --- |
| 5637 | Urinary bladder | ATCC | HTB-9 | Yes |
| HT-1197 | Urinary bladder | ATCC | CRL-1473 | Yes |
| HT-1376 | Urinary bladder | ATCC | CRL-1472 | Yes |
| RT4 | Urinary bladder | ATCC | HTB-2 | Yes |
| SW780 | Urinary bladder | ATCC | CRL-2169 | Yes |
| T-24 | Urinary bladder | ATCC | HTB-4 | Yes |
| TCCSUP | Urinary bladder | ATCC | HTB-5 | Yes |
| UM-UC-3 | Urinary bladder | ATCC | CRL-1749 | Yes |
| DU 145 | Prostate | ATCC | HTB-81 | Yes |
| LNCaP | Prostate | ATCC | CRL-1740 | Yes |
| NCI-H660 | Prostate | ATCC | CRL-5813 | Yes |
| PC-3 | Prostate | ATCC | CRL-1435 | Yes |
| 786-O | Renal | ATCC | CRL-1932 | Yes |
| ACHN | Renal | ATCC | CRL-1611 | Yes |
| Caki-1 | Renal | ATCC | HTB-46 | Yes |
| Caki-2 | Renal | ATCC | HTB-47 | Yes |

**Supplementary Table 9:** Overview of cell line authentication by STR profiling of the cancer cell lines included in the present study. The bladder cancer cell lines comprise the ATCC’s Bladder Cancer Cell Panel (ATCC® TCP-1020™), representing varying degree of genetic complexity.

| Package | Version | Available from | Reference | Comment |
| --- | --- | --- | --- | --- |
| FASTQC | 0.10.1 | http://www.bioinformatics.babraham.ac.uk/projects/fastqc/ |  |  |
| TrimGalore | 0.3.3 | www.bioinformatics.babraham.ac.uk/projects/trim_galore/ |  | In addition to the default parameters of the program, specific options for the quality of basecalls and paired end RRBS reads were specified:  (--phred64 --rrbs --paired) |
| Cutadapt | - |  | (1) |  |
| Bismark | 0.10.0 | https://www.bioinformatics.babraham.ac.uk/projects/bismark/ | (2) | Bismark: used with the default settings, except for specifying phred64 quality of basecalls.  Bismark methylation extractor: specific options to avoid bias during methylation scoring were included: (--no_overlap --ignore 1 --ignore_r2 3) |
| Bowtie2 | - |  | (3) |  |
| Samtools | 0.1.18 |  | (4) |  |
| MethylKit | 0.9.2 |  | (5) |  |
| SQLite studio | 3.0.5 |  |  |  |
| IGV | 2.3 |  | (6) |  |

**Supplementary Table 10: Software and parameters used in the bioinformatic pipeline for processing of the RRBS data and biomarker discovery.**

**References to Supplementary Table 10**

1. Martin M. Cutadapt removes adapter sequences from high-throughput sequencing reads. *EMBnetjournal.* 2011;17(1):10-12
2. Krueger F, Andrews SR. Bismark: a flexible aligner and methylation caller for Bisulfite-Seq applications. *Bioinformatics.* 2011;27(11):1571-1572
3. Langmead B, Salzberg SL. Fast gapped-read alignment with Bowtie 2. *Nat Methods.* 2012;9(4):357-359
4. Li H, Handsaker B, Wysoker A, et al. The Sequence Alignment/Map format and SAMtools. *Bioinformatics.* 2009;25(16):2078-2079
5. Akalin A, Kormaksson M, Li S, et al. methylKit: a comprehensive R package for the analysis of genome-wide DNA methylation profiles. *Genome Biol.* 2012;13(10):R87
6. Robinson JT, Thorvaldsdottir H, Winckler W, et al. Integrative genomics viewer. *Nat Biotechnol.* 2011;29(1):24-26

| Sample series | BlCa | NBM | PrCa | ReCa | Healthy controls | Hematuria patients with negative bladder cancer diagnosis | Total number of cases per series | Total number of samples per series |
| --- | --- | --- | --- | --- | --- | --- | --- | --- |
| Tissue series | 20 | 20 | 10 | 10 | - | - | 60 | 60 |
| Discovery urine series | 26 (19) | - | 18 (12) | 12 (8) | 56 (30) | - | 112 | 181 |
| Hematuria urine series | 93 (3) | - | - | - | - | 180 (35) | 273 | 311 |

**Supplementary Table 11: Number of clinical samples, both tissue and urine, analyzed in the present study.** In parentheses; the number of patients and controls with two parallel urine samples (see Supplementary Materials). Abbreviations: BlCa, bladder cancer; NBM, normal bladder mucosa; PrCa, prostate cancer; ReCa, renal cancer.

| Assay name | Sense primer | Antisense primer | Probe (unless otherwise specified; 6FAM-“sequence”-MGB) | Amplicon length | Ref |
| --- | --- | --- | --- | --- | --- |
| 1-26663001 | TTATTTTTGCGTGGGGGC | TCGTAACTACGAAAACTAAAACGCTAA | TTGTAGCGAGGCGAGGG | 142 | * |
| 1-234040601 | CGTACGTCGCGGAGGC | ACCCACCGAAAATCTACGCC | TTCGGGTATAGATCGCGC | 89 | * |
| 2-1747901 | CGAGCGTTAATAGGTAGCGGC | CCGTACGTCCGAACGTCC | TTATGGTCGACGGCGC | 83 | * |
| 3-197808301 | GGATCGTTAGCGCGTTTAATTG | CGACGACTACACAAAAACGAAAA | AGGACGTATTTTTCGTTTTA | 155 | * |
| 6-106958301 | TTTTTTTTTTGGGAGCGTTGC | ATCGCACGACTAAAACCCCA | ATTTTCGTATAGGTGGGTCGGT | 89 | * |
| 7-20824801 | GGAGTCGTTTTGCGAGTGC | AACAACGACGACGACGACG | AGGAGTCGTCGTCGTC | 63 | * |
| 7-65878301 | GTTGTGATATCGTCGAAGATACGC | CCTATAACCACCCGCCTACG | TTTCGGCGAACGTTC | 151 | * |
| 8-97173201 | GCGTCGAGTTGTGGGGC | TCGAAAAAAACCCTAACGCG | CGGGTCGTAAGCGT | 150 | * |
| 9-34665101 | GCGTCGAGTTGTGGGGC | TACGATACCGCGAACCCG | ATTCGATATTGGTATGAGGC | 121 | * |
| 9-94183601 | CGCGTTTTTACGCGGC | CTAACGCGAAAAATCCTCGC | CGTAGCGGGGTTTC | 91 | * |
| 9-117266901 | GGAGGACGAGTTTATCGATAGGTC | ATACCCGCGAAAACAACGAC | CGCGTTTATTTTTACGTCGAG | 111 | * |
| 10-102810001 | ATTCGATTCGTTTCGGATTTTC | ACGACGCGAAACAAACGAAC | AGGGGGCGTATCGC | 128 | * |
| 10-133796001 | GTTYGGGGCGTTCGTTC | CGCGCCTCCCTCGAAC | AGGTTCGAGTAGAGTCGA | 73 | * |
| 12-49690901 | TTAGTACGCGGATTTGTTCGAC | TCTAAATCTAACGTCGAAACTCGTTC | AATTACGAGGTTTTGCGTTAG | 95 | * |
| 12-54764201 | GAAAGTTAGCGTGTTTTGAAGCG | TCAACAATAAAAAAACGCGAACG | TTTTTTGGGTAAAGTTCGC | 132 | * |
| 12-124854101 | CGTTTGGGGGGTTCGTTC | AAACCRAACGAACGCTAAAAAAC | TTTATTTTGACGTATTTCGTTCG | 103 | * |
| 15-26108101 | TCGTTTTTGGTCGCGTTAGTC | AAACGACGAAAACGCCGA | TTGGAGCGTATCGTGC | 101 | * |
| 16-676701 | GTATGAACGCGGTTATGATGTACG | CGTTTTTATTTTTTGGTTAGCG | CGTTTTTATTTTTTGGTTAGCG | 79 | * |
| 16-67188901 | GGGATTTAATCGCGGATTTTC | ACGCCGAACGACTAAACGC | TTAAATAGCGTTTTTCGTTCG | 123 | * |
| 16-88600201 | TTGGGGGCGCGGTTAC | CGCGTCGTCCACGAAACTA | TTCGAGGACGGCGCG | 86 | * |
| 16-89267901 | TAGTGGGGCGCGGGT^**^ | CGCCTACTAACCTCCGCCTC | CGGTAAGTAGTTTTTCG | 95 | * |
| 16-89314201 | GGAGGGTAGTTGTTCGTTTTTTTC | CCCGAACTCCCGAACGAC | AGTTTTCGGTTGGTATTGC | 107 | * |
| 17-8055601 | GAGATCGGTTTTAGGATGGTCG | CCACTCGACTACGAACTAAAACGAC | TTTTTCGTACGTTTGTTCGTC | 96 | * |
| 17-38347601 | GGTAAGCGCGCGTAGTTGTT | CAAAAACGAACGACGACGC | AGAAGTTTATTAAGATCGCG | 112 | * |
| 17-40700201 | GTTGTTGATGCGTTTGGACG | CTACGAAATCGTCGACGAACAC | TGGTTTCGATTACGTCGTC | 83 | * |
| 19-15288601 | GCGGTCGCGTAGTTGGC | CAAACGCCCCGCGAA | CGGTAGGAGGGTTTC | 110 | * |
| 19-18118801 | TAGGGGCGTTTGTTATTTTGTTTTAC | GCCGCTTTTATACGACAAAACG | TAGAGTTACGTTTTTTGTCGCG | 132 | * |
| 22-27152801 | GCGCGCGCGGTTTAT | AACCGTAAAAAAACCCATTACCG | TTATCGCGCGGTCGC | 137 | * |
| VIM | TTCGGGAGTTAGTTCGCGTT | ACCGCCGAACATCCTACGA | TCGTCGTTTAGGTTATCGT | 108 | (1) |
| ALU | GGTTAGGTATAGTGGTTTATATTTGTAATTTTAGTA | ATTAACTAAACTAATCTTAAACTCCTAACCTCA | CCTACCTTAACCTCCC | 98 | (2) |
| EPHA3 | GGATTTATTAGGTGTGTAATGTTATGGATT | ACTCCACATAAATCTTCTAAACTAAATTCCT | 6VIC-TTGGTTGAGAATAAATTGGGTTT-MGB | 99 | (3) |
| KBTBD4 | TTTGTATGTGGTGGGAGGGTTT | ACAAAAAAACACACCACTCCCAA | 6VIC-TATGTGGAAGTGTAATAATG-MGB | 86 | (3) |
| PLEKHF1 | GTAGTTTTAGATGGTTTTTTGAGTTGGA | CACTCCCATCCTATCTTCCCTCTATA | 6VIC-AGGGATTAGAGTAGGTTTG-MGB | 100 | (3) |
| SYT10 | GAGGTAAATGTAGGTTTTTAGTGTTGATTTT | CTTTATCCTCCCAATACTAATTATTATTTCTCC | 6VIC-AGTATGGGTATAGAATTTGT-MGB | 94 | (3) |

*Assay designed for this study. **A rare SNP exists at the underlined position (G: 98.5% / A: 1.5%).

**Supplementary Table 12: Overview of all qMSP- and ddPCR assay sequences used in the present study.** Assay names reflect the chromosome number and the genomic start site of the window used for assay design (Supplementary Table 2). Primers were purchased from BioNordika Bergman and probes from Life Technologies.

**Reference list to Supplementary Table 12**

(1) Costa VL, Henrique R, Danielsen SA, et al. Three epigenetic biomarkers, GDF15, TMEFF2, and VIM, accurately predict bladder cancer from DNA-based analyses of urine samples. *Clin Cancer Res.* Dec 01 2010;16(23):5842-5851

(2) Weisenberger DJ, Campan M, Long TI, et al. Analysis of repetitive element DNA methylation by MethyLight. *Nucleic Acids Res.* 2005 2005;33(21):6823-6836

(3) Pharo HD, Andresen K, Berg KCG, Lothe RA, Jeanmougin M, Lind GE. A robust internal control for high-precision DNA methylation analyses by droplet digital PCR. *Clin Epigenetics.* 2018;10:24

| **ITEM TO CHECK** | **PROVIDED** | **COMMENT** | |  |  |
| --- | --- | --- | --- | --- | --- |
| **Column1** | **Y/N** | **Column2** | |  |  |
| **1. SPECIMEN** |  |  | |  |  |
| Detailed description of specimen type and numbers | **Y** | Supplementary Materials & Supplementary Table 11 | |  |  |
| Sampling procedure (including time to storage) | **Y** | Materials and Methods in the main text & Supplementary Methods | |  |  |
| Sample aliquotation, storage conditions and duration | **Y** | Discovery series urine: urine pellets stored at -80°C for up to 6 months. Hematuria series: urine filters stored at room temperature for several months (as previously described by Dahmcke et al. Eur Urol. 2016) | |  |  |
| **2. NUCLEIC ACID EXTRACTION** |  |  | |  |  |
| Description of extraction method including amount of sample processed | **Y** | Supplementary Methods | |  |  |
| Volume of solvent used to elute/resuspend extract | **Y** | Discovery series urine: 60 µl + 20 µl AE-buffer (QIAamp DNA Mini Kit; Qiagen). Hematuria series: 50 µl 1xTE buffer (10mM Tris-HCl, pH 8.0, 1mM ethylenediaminetetraacetic acid) | |  |  |
| Number of extraction replicates | **Y** | None | |  |  |
| Extraction blanks included? | **Y** | Not performed | |  |  |
| **3. NUCLEIC ACID ASSESSMENT AND STORAGE** |  |  | |  |  |
| Method to evaluate quality of nucleic acids | **Y** | NanoDrop 1000 Spectrophotometer (Thermo Fisher Scientific) | |  |  |
| Method to evaluate quantity of nucleic acids (including molecular weight and calculations when using mass) | **Y** | NanoDrop 1000 Spectrophotometer (Thermo Fisher Scientific) | |  |  |
| Storage conditions: temperature, concentration, duration, buffer, aliquots | **Y** | Discovery series urine: Storage at 4°C, 6-216 ng/µl, months, in AE-buffer (QIAamp DNA Mini Kit; Qiagen). Hematuria series: Storage at -80°C, 0.006-186 ng/µl, months, in 1xTE buffer (10mM Tris-HCl, pH 8.0, 1mM ethylenediaminetetraacetic acid). | |  |  |
| Clear description of dilution steps used to prepare working DNA solution | **Y** | Discovery series: If DNA concentration as measured by NanoDrop exceeded 200 ng/µl, the sample was diluted down to ~200ng/µl in AE-buffer (QIAamp DNA Mini Kit; Qiagen). Hematuria series: No dilution performed. | |  |  |
| **4. NUCLEIC ACID MODIFICATION** | **Y** |  | |  |  |
| Template modification (digestion, sonication, pre-amplification, bisulphite etc.) | **Y** | Bisulfite conversion, Supplementary Methods | |  |  |
| Details of repurification following modification if performed | **Y** | Not performed | |  |  |
| **5. REVERSE TRANSCRIPTION** | **NA** | All templates measured with ddPCR were DNA templates | |  |  |
| cDNA priming method and concentration | **N** | N/A | |  |  |
| One or two step protocol (include reaction details for two step) | **N** | N/A | |  |  |
| Amount of RNA added per reaction | **N** | N/A | |  |  |
| Detailed reaction components and conditions | **N** | N/A | |  |  |
| Estimated copies measured with and without addition of RT* | **N** | N/A | |  |  |
| Manufacturer of reagents used with catalogue and lot numbers | **N** | N/A | |  |  |
| Storage of cDNA: temperature, concentration, duration, buffer and aliquots | **N** | N/A | |  |  |
| **6. dPCR OLIGONUCLEOTIDES DESIGN AND TARGET INFORMATION** |  |  | |  |  |
| Sequence accession number or official gene symbol | **Y** | Supplementary Table 2 | |  |  |
| Method (software) used for design and *in silico* verification | **Y** | Primer Express 3.0 (Applied Biosystems) | |  |  |
| Location of amplicon | **Y** | Supplementary Table 2 | |  |  |
| Amplicon length | **Y** | Supplementary Table 12 | |  |  |
| Primer and probe sequences (or amplicon context sequence)** | **Y** | Supplementary Table 12 | |  |  |
| Location and identity of any modifications | **Y** | None | |  |  |
| Manufacturer of oligonucleotides | **Y** | Supplementary Table 12 | |  |  |
| **7. dPCR PROTOCOL** |  |  | |  |  |
| Manufacturer of dPCR instrument and instrument model | **Y** | QX200™ Droplet Digital™ PCR System (BioRad) | |  |  |
| Buffer/kit manufacturer with catalogue and lot number | **Y** | All reagents, instruments and equipments used are those recommended from the manufacturer (BioRad). **1x ddPCR Supermix for Probes** (Cat.No. 186-3023): Lot numbers 64091066, 64094322, 64164638, 64191911, 64209288, 64274891. **Droplet Generation Oil for Probes** (Cat.No. 186-3005) and **Automated Droplet Generation Oil for Probes** (Cat.No. 186-4110): Lot numbers 11222016A, 64052956, 64052954, 64109503, 18G3056089, 18G3056098, 54275481. **ddPCR Droplet Reader Oil** (Cat.No. 186-3004): Lot numbers 64049253, 64081870, 64086321, 64090200, 64109506, MW181307019, MW18G16567. | |  |  |
| Primer and probe concentration | **Y** | Supplementary Methods | |  |  |
| Pre-reaction volume and composition (incl. amount of template and if restriction enzyme added) | **Y** | Supplementary Methods | |  |  |
| Template treatment (initial heating or chemical denaturation) | **Y** | None | |  |  |
| Polymerase identity and concentration, Mg++ and dNTP concentrations*** | **Y** | Polymerase contained in the 1x ddPCR Supermix for Probes (BioRad; Cat.No. 186-3023) | |  |  |
| Complete thermocycling parameters | **Y** | Supplementary Table 17 | |  |  |
| **8. ASSAY VALIDATION** |  |  | |  |  |
| Details of optimisation performed | **Y** | Assays were quality controlled on both the qMSP and the ddPCR platform with standardcurve, positive and negtive controls. More information is available upon reasonable request. | |  |  |
| Analytical specificity (vs. related sequences) and limit of blank (LOB) | **Y** | Analytic specificity (vs. related sequences): Assay specificities were ensured by lack of amplification of non-bisulfite converted DNA (unpublished data, available upon reasonable request).  LOB was determined as described by Armbruster et al. Clin. Biochem. Rev. 2008; **LoB = mean of blanks + 1.645x(SD of blanks)**. LOB was calculated based on an average of 40 blank reactions per assay: For assay 6-106958301, LOB = 0.11 cop/µl. For the remaining assays, LOB = 0.00 cop/µl. | |  |  |
| Analytical sensitivity/LoD and how this was evaluated | **Y** | The LoD for each assay was evaluated visually based on a titration curve consisting of two-fold dilutions of a methylated human DNA standard (Zymo Research; 5ng-0.08ng). Nine replicates were included for each of the seven titration points. Evaluation of the coefficient of variation of the target concentration (in cop/µl) for each titration point supported censoring reactions with 1-2 positive droplets for all assays (data available upon reasonable request). | |  |  |
| Testing for inhibitors (from biological matrix/extraction) | **Y** | Not performed | |  |  |
| **9. DATA ANALYSIS** |  |  | |  |  |
| Description of dPCR experimental design | **Y** | Supplementary Methods | |  |  |
| Comprehensive details negative and positive of controls (whether applied for QC or for estimation of error) | **Y** | Supplementary Methods | |  |  |
| Partition classification method (thresholding) | **Y** | PoDCall algorithm (https://github.com/HansPetterBrodal/PoDCall; Pharo et al. Clin Epigenetics. 2018) | |  |  |
| Examples of positive and negative experimental results (including fluorescence plots in supplemental material) | **Y** | Supplementary Figure 6 | |  |  |
| Description of technical replication | **Y** | 12 replicates of two representative urine samples were run for all assays | |  |  |
| Repeatability (intra-experiment variation) | **Y** | The average coefficient of variation for the two urine samples with 12 replicates were calculated per assay: 6-106958301=0.08, 9-94183601=0.11, 19-18118801=0.08, 19-15288601=0.11, 16-89314201=0.06, 16-89267901=0.10, 9-117266901=0.25, VIM=0.08 | |  |  |
| Reproducibility (inter-experiment/user/lab etc. variation) | **Y** | Consistent concentrations were observed for the positive-methylation control (methylated human DNA standard; Zymo Research) for each assay across all experiments (data available upon reasonable request). | |  |  |
| Number of partitions measured (average and standard deviation) | **Y** | Number of partitions: Average = 14061 droplets, Standard deviation = 2580 droplets | |  |  |
| Partition volume | **Y** | Average: 0.834 nL (inherent of the Bio-Rad Droplet Generator; Corbisier et al. Anal Bioanal Chem. 2015) | |  |  |
| Copies per partition (λ or equivalent) (average and standard deviation) | **Y** | λ= –ln(1–*k/n*), where *k* = number of positive partitions, and *n* = number of partitions. Assay=Average λ/ Standard deviation λ; 6-106958301=0.007/0.033, 9-94183601=0.001/0.006, 19-18118801=0.002/0.011, 19-15288601=0.003/0.009, 16-89314201=0.003/0.012, 16-89267901=0.002/0.009, 9-117266901=0.001/0.006, VIM=0.002/0.011 | |  |  |
| dPCR analysis program (source, version) | **Y** | QuantaSoft version 1.7.4.0917 (BioRad) | |  |  |
| Description of normalisation method | **Y** | 4Plex control; Supplementary Methods | |  |  |
| Statistical methods used for analysis | **Y** | Supplementary Methods | |  |  |
| Data transparency | raw data available on request: | Raw data is available upon reasonable request | |  |  |
| *Assessing the absence of DNA using a no RT assay (or where RT has been inactivated) is essential when first extracting RNA. Once the sample has been validated as DNA-free, inclusion of a no-RT control is desirable, but no longer essential. ** Disclosure of the primer and probe sequence is highly desirable and strongly encouraged. However, since not all commercial pre-designed assay vendors provide this information when it is not available assay context sequences must be submitted (Bustin et al. Primer sequence disclosure: A clarification of the miqe guidelines. Clin Chem 2011;57:919-21.) *** Details of reaction components is highly desirable, however not always possible for commercial disclosure reasons. Inclusion of catalogue number is essential where component reagent details are not available | | |  | |  |

**Supplementary Table 13:** The ddPCR analyses were performed according to the dMIQE2020 guidelines.

|  | **Section & Topic** | **No** | **Item** | **Reported where?** |
| --- | --- | --- | --- | --- |
|  |  |  |  |  |
|  | **TITLE OR ABSTRACT** |  |  |  |
|  |  | **1** | Identification as a study of diagnostic accuracy using at least one measure of accuracy  (such as sensitivity, specificity, predictive values, or AUC) | Abstract |
|  | **ABSTRACT** |  |  |  |
|  |  | **2** | Structured summary of study design, methods, results, and conclusions  (for specific guidance, see STARD for Abstracts) | Abstract |
|  | **INTRODUCTION** |  |  |  |
|  |  | **3** | Scientific and clinical background, including the intended use and clinical role of the index test | Background |
|  |  | **4** | Study objectives and hypotheses | Background |
|  | **METHODS** |  |  |  |
|  | *Study design* | **5** | Whether data collection was planned before the index test and reference standard  were performed (prospective study) or after (retrospective study) | 1. Supplementary Materials/1.1. Clinical samples |
|  | *Participants* | **6** | Eligibility criteria | 1. Supplementary Materials/1.1. Clinical samples |
|  |  | **7** | On what basis potentially eligible participants were identified  (such as symptoms, results from previous tests, inclusion in registry) | 1. Supplementary Materials/1.1. Clinical samples |
|  |  | **8** | Where and when potentially eligible participants were identified (setting, location and dates) | 1. Supplementary Materials/1.1. Clinical samples |
|  |  | **9** | Whether participants formed a consecutive, random or convenience series | 1. Supplementary Materials/1.1. Clinical samples |
|  | *Test methods* | **10a** | Index test, in sufficient detail to allow replication | Methods/“Analysis of the biomarker panel in urine – discovery and hematuria series” & Supplementary Methods |
|  |  | **10b** | Reference standard, in sufficient detail to allow replication | Methods/“Analysis of the biomarker panel in urine – discovery and hematuria series” |
|  |  | **11** | Rationale for choosing the reference standard (if alternatives exist) | Not relevant |
|  |  | **12a** | Definition of and rationale for test positivity cut-offs or result categories  of the index test, distinguishing pre-specified from exploratory | Results/” BladMetrix performance in urine – the discovery series”, Supplementary Methods, Supplementary Table 5 |
|  |  | **12b** | Definition of and rationale for test positivity cut-offs or result categories  of the reference standard, distinguishing pre-specified from exploratory | Not relevant |
|  |  | **13a** | Whether clinical information and reference standard results were available  to the performers/readers of the index test | Results/” BladMetrix performance among hematuria patients – a blinded analysis of a prospectively collected urine series” |
|  |  | **13b** | Whether clinical information and index test results were available  to the assessors of the reference standard | Not relevant |
|  | *Analysis* | **14** | Methods for estimating or comparing measures of diagnostic accuracy | Methods/”Statistics” |
|  |  | **15** | How indeterminate index test or reference standard results were handled | Methods/”Statistics” |
|  |  | **16** | How missing data on the index test and reference standard were handled | Supplementary Methods/” Targeted DNA methylation analyses: quantitative methylation-specific PCR (qMSP) and droplet digital PCR (ddPCR)” |
|  |  | **17** | Any analyses of variability in diagnostic accuracy, distinguishing pre-specified from exploratory | Not relevant |
|  |  | **18** | Intended sample size and how it was determined | Not relevant |
|  | **RESULTS** |  |  |  |
|  | *Participants* | **19** | Flow of participants, using a diagram | Not relevant |
|  |  | **20** | Baseline demographic and clinical characteristics of participants | Table 3 |
|  |  | **21a** | Distribution of severity of disease in those with the target condition | Table 3 |
|  |  | **21b** | Distribution of alternative diagnoses in those without the target condition | Not relevant |
|  |  | **22** | Time interval and any clinical interventions between index test and reference standard | Not relevant |
|  | *Test results* | **23** | Cross tabulation of the index test results (or their distribution)  by the results of the reference standard | Supplementary Table 6 |
|  |  | **24** | Estimates of diagnostic accuracy and their precision (such as 95% confidence intervals) | Table 1 |
|  |  | **25** | Any adverse events from performing the index test or the reference standard | Not relevant |
|  | **DISCUSSION** |  |  |  |
|  |  | **26** | Study limitations, including sources of potential bias, statistical uncertainty, and generalisability | Discussion |
|  |  | **27** | Implications for practice, including the intended use and clinical role of the index test | Discussion |
|  | **OTHER INFORMATION** |  |  |  |
|  |  | **28** | Registration number and name of registry | Not relevant |
|  |  | **29** | Where the full study protocol can be accessed | From the authors upon reasonable request |
|  |  | **30** | Sources of funding and other support; role of funders | Declarations |
|  |  |  |  |  |

**Supplementary Table 14: The Standards for Reporting of Diagnostic Accuracy Studies (STARD) checklist.**

| Primer | Sequence |
| --- | --- |
| 3’ | AGATCGGAAGAGCACACGTCTGAACTCCAGTCAC |
| 5’ | AGATCGGAAGAGCGTCGTGTAGGGAAAGAGTGTA |

**Supplementary Table 15: Illumina adapter sequences used in the RRBS.**

| Cancer cell line | C’s methylated in non-CpG context (CHH) | Bisulfite conversion rate |
| --- | --- | --- |
| 5637 | 0.4 | 99.6 |
| HT-1197 | 0.4 | 99.6 |
| HT-1376 | 0.4 | 99.6 |
| RT4 | 0.4 | 99.6 |
| SW780 | 0.5 | 99.5 |
| T-24 | 0.4 | 99.6 |
| TCCSUP | 0.4 | 99.6 |
| UM-UC-3 | 0.5 | 99.5 |
| DU 145 | 0.5 | 99.5 |
| LNCaP | 0.4 | 99.6 |
| NCI-H660 | 0.5 | 99.5 |
| PC-3 | 0.4 | 99.6 |
| 786-O | 0.5 | 99.5 |
| ACHN | 0.5 | 99.5 |
| Caki-1 | 0.4 | 99.6 |
| Caki-2 | 0.5 | 99.5 |

**Supplementary Table 16: Bisulfite conversion rate.** Data has been extracted based on the report from Bismark.

| C**ycling step** | **Temperature, °C** | **Time** | **Ramp rate** | **Number of cycles** |
| --- | --- | --- | --- | --- |
| Enzyme activation | 95 | 10 min | 2°C/sec | 1 |
| Denaturation | 94 | 30 sec |  | 40 |
| Annealing/extension | 60 | 1 min |  | 40 |
| Enzyme deactivation | 98 | 10 min |  | 1 |
| Hold | 4 | Infinite |  | 1 |

Supplementary Table 17: The PCR thermal cycling conditions for the ddPCR experiments. The conditions are recommended from the manufacturer.

| Assay name | Threshold for methylation scoring (methylated copies/µl) |
| --- | --- |
| 6-106958301 | 7.33 |
| 9-94183601 | 0.170 |
| 9-117266901 | 0.352 |
| 16-89267901 | 0.197 |
| 16-89314201 | 0.310 |
| 19-15288601 | 4.027 |
| 19-18118801 | 0.840 |
| VIM | 0.405 |

**Supplementary Table 18: Assay-specific thresholds for methylation scoring.** For each biomarker, the threshold for methylation scoring is defined as the value with highest sensitivity with a specificity ≥95% based on ROC curve analyses.
